# Quantitative microscopy of the *Drosophila* ovary shows multiple niche signals specify progenitor cell fate

**DOI:** 10.1038/s41467-017-01322-9

**Published:** 2017-11-01

**Authors:** Wei Dai, Amy Peterson, Thomas Kenney, Haley Burrous, Denise J. Montell

**Affiliations:** MCDB Department, University of California, Santa Barbara, CA 93106 USA

## Abstract

Adult stem cells commonly give rise to transit-amplifying progenitors, whose progeny differentiate into distinct cell types. It is unclear if stem cell niche signals coordinate fate decisions within the progenitor pool. Here we use quantitative analysis of Wnt, Hh, and Notch signalling reporters and the cell fate markers Eyes Absent (Eya) and Castor (Cas) to study the effects of hyper-activation and loss of niche signals on progenitor development in the *Drosophila* ovary. Follicle stem cell (FSC) progeny adopt distinct polar, stalk, and main body cell fates. We show that Wnt signalling transiently inhibits expression of the main body cell fate determinant Eya, and Wnt hyperactivity strongly biases cells towards polar and stalk fates. Hh signalling independently controls the proliferation to differentiation transition. Notch is permissive but not instructive for differentiation of multiple cell types. These findings reveal that multiple niche signals coordinate cell fates and differentiation of progenitor cells.

## Abstract (web summary)

In the *Drosophila* ovary, it is unclear how signalling from the stem cell niche regulates follicle precursor cell fate/differentiation. Here, the authors use quantitative microscopy to define the role of Wnt, Hedgehog and Notch signalling in progenitor cells, showing regulation of cell fate and differentiation.

## Introduction

Adult stem cells are important for tissue homoeostasis and regeneration due to their ability to both self-renew and generate multiple types of differentiated daughters. Adult stem cells are located in a niche that provides the proper microenvironment to maintain “stemness”^1, 2^. The progeny of stem cells that move away from the niche generally go through a precursor cell (or progenitor cell, transit-amplifying cell) stage before they differentiate^3, 4^. However, it is unclear whether the precursor state is simply a loss of stemness due to displacement from niche signals, or whether secreted niche factors might act as graded morphogens that establish distinct cell fates at different concentrations and distances from the niche.

**Fig. 1:**
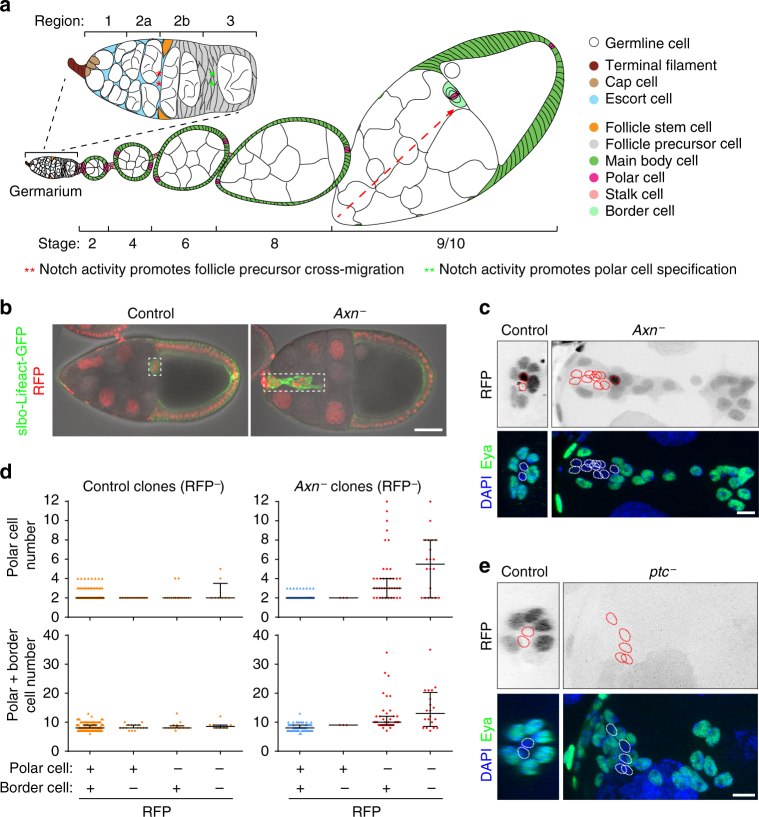
*Axn* mutant clones cause supernumerary polar cells. **a** Drawing of a *Drosophila* ovariole in the sagittal view. Dashed arrow indicates the border cell migration path. **b** Sagittal view of stage 10 egg chambers with control (left panel) or FRT82B, *Axn* ^*1511*^ mosaic (right panel) border cell clusters (dashed boxes). Scale bar, 50 μm. **c** 3D projection view of border cell clusters containing FRT82B control or FRT82B, *Axn* ^*1511*^ mosaic clones. Homozygous mutant cells are RFP_−_negative (RFP^−^). Polar cells are identified by absence of Eya expression (dotted circles). Scale bar, 10 μm. **d** Quantification of all border cell clusters in stage 9/10 egg chambers, regardless of whether they have clones or not, in FRT82B control or FRT82B, *Axn* ^*1511*^, 4-5 days after clone induction. Data from *n* = 284 egg chambers for control, 222 for *Axn* ^*−*^. Each dot represents one border cell cluster. Lines show the median with interquartile range. **e** Border cell cluster in FRT42D control or FRT42D, *ptc* ^*S2*^ mutant clones. Homozygous mutant cells are RFP^−^. Polar cells are Eya^−^ (dotted circles). Scale bar, 10 μm

The *Drosophila* ovary is an appealing model for studying adult stem cells^5^. Each ovary contains 16–20 ovarioles, which are chains of egg chambers in increasing stages of maturity^6^ (Fig. 1a). Development begins in the germarium, which is located at the anterior tip of the ovariole. The anterior half of the germarium, region 1, contains germline stem cells and their progeny, which continue dividing to produce 16-cell cysts. Somatic escort cells surround the developing cysts as they progress to region 2a. The FSCs are located at the region 2a/2b boundary^7^, where cysts exchange their escort cell covering for the FSC daughters. The posterior half of the germarium contains flattened cysts in region 2b, followed by rounded region 3 cysts. Follicle precursor cells associate with region 2b and region 3 cysts, and their progeny adopt distinct polar, stalk, and main body cell fates, which serve different functions. However, the molecular mechanisms that govern these earliest cell fate decisions are mostly unknown and most precursors in region 2b and region 3 do not yet express mature cell fate markers^8–10^.

Several signalling pathways have been implicated in regulating follicle precursor cell fate specification and differentiation. Notch signalling is required for polar cell specification^9^ and is present in mature polar cells at high levels in region 3/stage 1^11^. Earlier Notch activity at the region 2a/2b boundary is required for one FSC daughter to migrate laterally across the germarium, while other daughters move posteriorly^8^. However, Notch activity is not sufficient to induce ectopic polar cells in the main body region^10, 12^, suggesting that additional factors control polar cell fate.

Escort cells form the FSC niche^2, 13, 14^. Niche factors important for FSC maintenance include Wnt, Hh, epidermal growth factor (EGF), and bone morphogenetic protein, which are crucial in many adult stem cell niches^13, 15–20^. Hyper-activation of Wnt or Hh signalling causes defects in follicle cell differentiation^15, 21^, but the origins of these phenotypes are not understood and it remains unclear whether these niche signals normally regulate progenitor cell fate or differentiation.

In a forward genetic screen for mutations that disrupt cell fates in the ovary, we identified a mutant allele of *Axin* (*Axn*), a negative regulator in the Wnt pathway. The phenotype resembled that caused by mutations in *patched* (*ptc*) or *costal* (*cos*), two negative regulators of Hh signalling. However, when we traced both defects back to the earliest steps of follicle cell specification, we found differences. We developed quantitative analyses of the differentiation markers, Eya and Cas, as well as Wnt, Hh and Notch signalling reporters, which revealed independent roles for these three pathways. We found that the Wnt and Hh responses exhibited distinct signal patterns in the germarium. Wnt signalling transiently suppressed the main body cell fate factor Eya, whereas Hh signalling delayed differentiation of all follicle cell types. Loss of negative regulators caused more severe phenotypes than loss of positive regulators. Both loss and gain of function of the two pathways produced additive phenotypic effects. Notch knockdown caused multiple cell differentiation defects, but constitutively active Notch was not instructive for any particular cell fate. We conclude that combinatorial signalling produces the appropriate spatial patterning of cell types and temporal patterning of differentiation.

## Results

### Distinct effects of Wnt and Hh hyper-activation

At stage 8 of oogenesis, anterior polar cells specify neighbouring epithelial follicle cells as motile border cells, and together they migrate as a cluster during stage 9 (Fig. 1a). In a forward genetic screen of EMS-induced mutations that cause border cell defects in mosaic clones^22^, we identified a line that produced abnormally large border cell clusters. Compared to control clusters, which are usually composed of 5–7 migratory cells surrounding two polar cells, clusters containing mutant cells showed as many as 6–12 polar cells and 14–35 total cells per cluster (Fig. 1b–d). The phenotype was autonomous to the polar cells, as supernumerary polar cells and over-sized border cell clusters were only observed when polar cells were homozygous for the mutation (Fig. 1d). The supernumerary polar cell phenotype resembled those previously reported for *eya* ^23^, *cos* ^24^, and *ptc* (Fig. 1e ^21^). We mapped the new mutation to genomic location 99D3 (Supplementary Fig. 1a−c), which contains the *Axn* gene. *Axn* allele S044230 produced a similar phenotype (Supplementary Fig. 2) and failed to complement the new mutation for lethality. We therefore named the new allele *Axn* ^*1511*^. This supernumerary polar cell phenotype was somewhat surprising, as it had not previously been reported for the *Axn* gene.

**Fig. 2:**
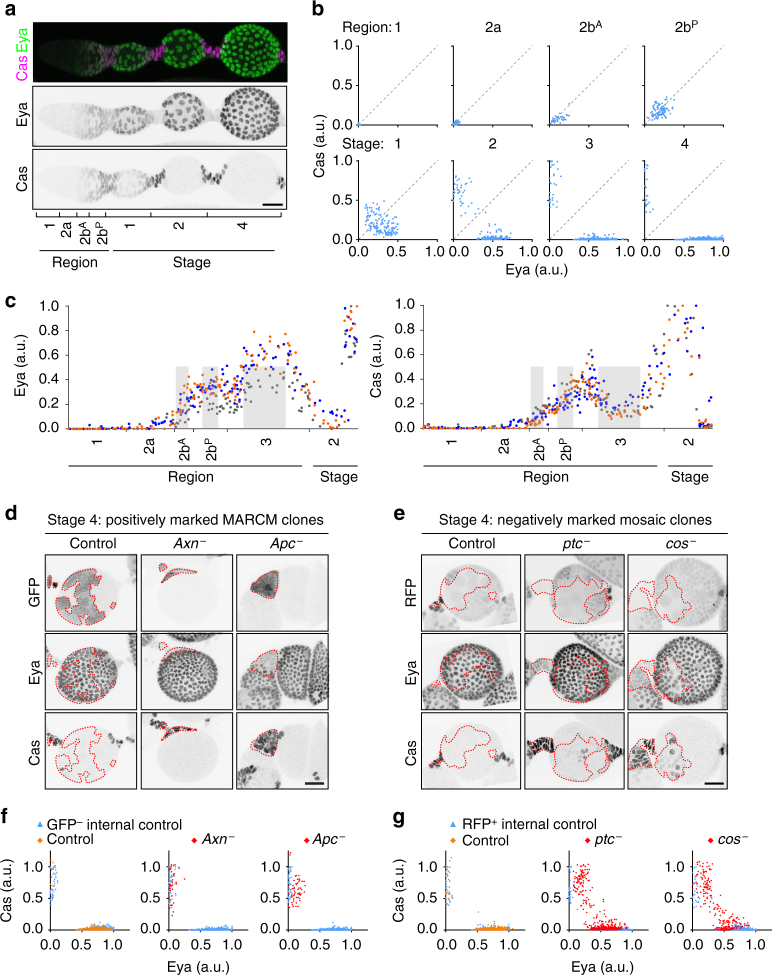
Differential effects of Wnt and Hh hyper-activation on follicle cell differentiation. **a** 3D projection view of one half of a wild type ovariole from the germarium to stage 4, stained with Eya (green) and Cas (magenta) antibodies. Individual channels are shown in black and white. **b** Quantification of Eya and Cas fluorescence intensity in all somatic cells in germarium regions 1–3 and in stage 1–4 egg chambers. Data from *n* = 2122 cells from 3 ovarioles per stage. Each dot represents one cell. Data were normalized to maximum Eya or Cas intensity per ovariole. **c** Quantification of Eya and Cas intensity in all somatic cells in germarium until anterior of stage 2 along the anterior–posterior axis. Data from *n* = 399 cells from three ovarioles. Grey bars indicate position of the DNA from the germline cysts. Different colours represent different ovarioles. Data were normalized to maximum Eya and Cas intensity per sample. **d** Stage 4 egg chambers with FRT82B control mosaic FSC clones compared to FRT82B, *Axn* ^*1511*^, or FRT82B, *Apc2* ^*g10*^, *Apc* ^*Q8*^. Homozygous mutant cells in the main body and anterior polar/stalk regions are outlined (GFP^+^, dashed lines). Eya^+^ GFP^−^ cells appear in the outlined *Apc* ^−^ clone due to *Z* stack projection. **e** Stage 4 egg chambers with FRT42D control, FRT42D, *ptc* ^*S2*^ or FRT42D, *cos* ^*H29*^ mosaic FSC clones. Homozygous mutant cells are RFP^−^ (dashed lines). **f**, **g** Quantification of Eya and Cas fluorescence intensity in mosaic FSC clones. Data from *n* = 1222–1446 cells from 4 stage 4 egg chambers per genotype. Data were normalized to maximum Eya or Cas intensity in internal control cells per egg chamber. Scale bars, 20 μm. a.u., arbitrary unit

In addition to supernumerary polar cells, *Axn* ^−^ and *ptc* ^−^ clones showed abnormal stalks, consistent with previous reports^15, 25^. Polar and stalk cell specification occurs early in ovarian development, and these cells stop dividing soon after they exit the germarium^7, 26^. In the ovary, hyperactive Wnt or Hh signalling affects differentiation^15, 21^. To ask what aspects of differentiation were affected by Wnt or Hh hyper-activation, we used two complementary markers, Eya^23^ and Cas^27^ (Fig. 2a). To be comprehensive, we performed 3D reconstructions of ovarioles and quantified the levels of both Eya and Cas in every somatic cell (Fig. 2b; Supplementary Fig. 3; Supplementary Movie 1). We found barely detectable levels of either protein in regions 1 and 2a escort cells. Low levels of both Eya and Cas were present in FSCs which increased in their daughters in anterior and posterior region 2b (2b^A^ and 2b^P^). Eya and Cas showed differential expression in region 3/stage 1, such that some cells expressed higher levels of one or the other (indicated by divergence from the diagonal in the graphs in Fig. 2b). As development proceeded through stages 1–4, the levels of Eya and Cas diverged more and more. By stage 4, Eya and Cas became completely distinct markers for main body (Eya^+^ Cas^−^) versus polar or stalk (Eya^−^ Cas^+^) fates (Fig. 2a, b). Further detailed examination revealed that the Eya level increased precipitously within region 2b^A^ along the anterior–posterior axis, while the Cas level increased more gradually throughout the 2b^A^ and 2b^P^ regions (Fig. 2c; Supplementary Fig. 4). The combination of Eya and Cas distinguished cells in region 2b, where both markers are low but increasing from those in region 3/stage 1, which began to diverge into Eya^low^ Cas^high^ and Eya^high^ Cas^low^ cells though many cells still express both to some degree. Thus, Eya and Cas are excellent markers for studying the earliest cell fates in the ovary.

To characterize the defects caused by hyperactive Wnt or Hh signalling more precisely, we made FSC clones and stained them for Eya and Cas (Fig. 2d, e). Hyperactive Wnt signalling, caused by loss of the destruction complex component *Axn* ^−^ or *adenomatous polyposis coli* (*Apc* ^−^) produced many egg chambers containing only Eya^−^ Cas^+^ clones, in contrast to controls in which Eya^+^ cells were frequent (Fig. 2d, f). By contrast, FSC clones with hyperactive Hh signalling caused by loss of the negative regulators *ptc* ^−^ or *cos* ^−^ were not biased towards polar/stalk fates or terminal positions. They instead produced many Eya^+^ Cas^+^ cells in stage 4 that resembled control cells in stages 1 and 2. Eya^+^ Cas^+^ cells were virtually never observed in controls in stage 4 (Fig. 2b, e, g). These results suggested distinct responses to Wnt or Hh hyper-activation.

### Wnt and Hh act independently in the germarium

**Fig. 3:**
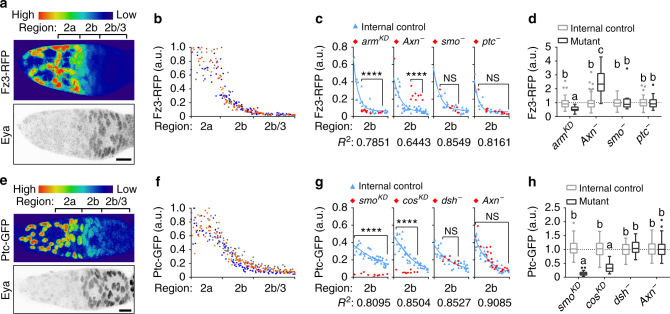
Independent actions of Wnt and Hh in the germarium. **a** 3D projection view of one half of a germarium expressing the Wnt activity reporter fz3*-RFP* and stained for Eya. RFP intensity is displayed using the “Physics” lookup table. **b** Quantification of Wnt reporter intensity in all somatic cells from region 2-3 along the anterior–posterior axis. Data from *n* = 276 cells from 3 germaria. Each dot represents one cell. The three colours represent the three different germaria. **c** Wnt reporter activity in germaria with *armRNAi*, *Axn* ^*S044230*^, *smo* ^*D16*^, or *ptc* ^*S2*^ mutant clones (red diamonds) compared to control cells in the same germarium (blue triangles). *R* ^2^ quantifies goodness of fit of nonlinear regression. **d** Quantification of Wnt reporter activity in germaria region 2b with mosaic clones. Data (median with interquartile range) from *n* = 16–103 cells from 3 germaria per genotype. Data were normalized to the predicted value on the one phase decay curve fitted on the internal control cells. *P* < 0.01. **e** A germarium expressing the Hh activity reporter *ptc-GFP*. **f** Quantification of Hh reporter intensity in all somatic cells from regions 2 to 3 along the anterior–posterior axis. Data from *n* = 314 cells from 3 germaria. **g** Hh reporter activity in germaria with *smoRNAi*, *cosRNAi*, *dsh* ^*3*^, or *Axn* ^*S044230*^ mutant clones. **h** Quantification of Hh reporter activity in germarium region 2b with mosaic clones. Data (median with interquartile range) from *n* = 18–105 cells from 3 germaria per genotype. *P* < 0.0001. Scale bars, 10 μm. **P < 0.01; ****P < 0.0001 (Mann–Whitney test). Samples labelled with different letters are significantly different (Kruskal–Wallis with Dunn’s test)

Wnt and Hh signalling positively regulate one another in some settings^28^, while they antagonize^29, 30^ or play independent roles^31^ in other cases. In the ovary, Wnt and Hh are stem cell niche factors produced in cap cells and escort cells^13, 19, 20, 32^. To understand their relationship, we examined Wnt and Hh activity patterns. We used *frizzled 3* (*fz3*)-*RFP* ^33, 34^, which was previously verified as the best available reporter for Wnt signalling activity in the germarium as it is most consistent with the expression patterns of Wnt ligands^34^. Fz3-RFP was highly expressed in regions 1–2a and showed a graded pattern in region 2b (Fig. 3a, b). To verify the fidelity of the reporter, we performed mosaic analysis. Reducing expression of the positive regulator of Wnt signalling β-catenin (in *Drosophila* Armadillo (Arm)) by RNAi reduced the Fz3-RFP signal and Fz3-RFP increased in *Axn* ^−^ clones, demonstrating that it is indeed responsive to Wnt signalling (Fig. 3c, d; Supplementary Fig. 5a). The level of Fz3-RFP expression present in *Axn* ^−^ cells in region 2b^P^ was about ~30% of the maximum endogenous level, found in escort cells, and ~75% of the level normally found in region 2b^A^, thus well within the physiological range.

To decipher the relationship between Wnt and Hh signalling, we examined the pattern of *fz3-RFP* in *smo* ^−^ or *ptc* ^−^ clones. Changing Hh signalling had no detectable effect on the Wnt reporter in region 2b (Fig. 3c, d).* ptc*-*GFP* is a reporter for Hh signalling^13^ and shows a pattern similar to *fz3-RFP* (Fig. 3e, f), though the signal extends more posteriorly. The Ptc-GFP signal was reduced when knocking down *smoothened* (*smo*), a positive regulator in the Hh pathway (Fig. 3g, h; Supplementary Fig. 5b). Unexpectedly, *cosRNAi* also caused a reduction of Ptc-GFP signal in region 2b (Fig. 3g, h; Supplementary Fig. 5c), while in later stages the signal increased as expected for loss of a negative regulator (Supplementary Fig. 6). The pattern of Ptc-GFP in *dishevelled* (*dsh*, a positive regulator in the Wnt pathway) or *Axn* mutant clones was not measurably different from control clones. (Fig. 3g, h). Thus, Wnt and Hh appear to function independently in the ovary.

### Wnt signalling inhibits expression of the main body factor Eya

**Fig. 4:**
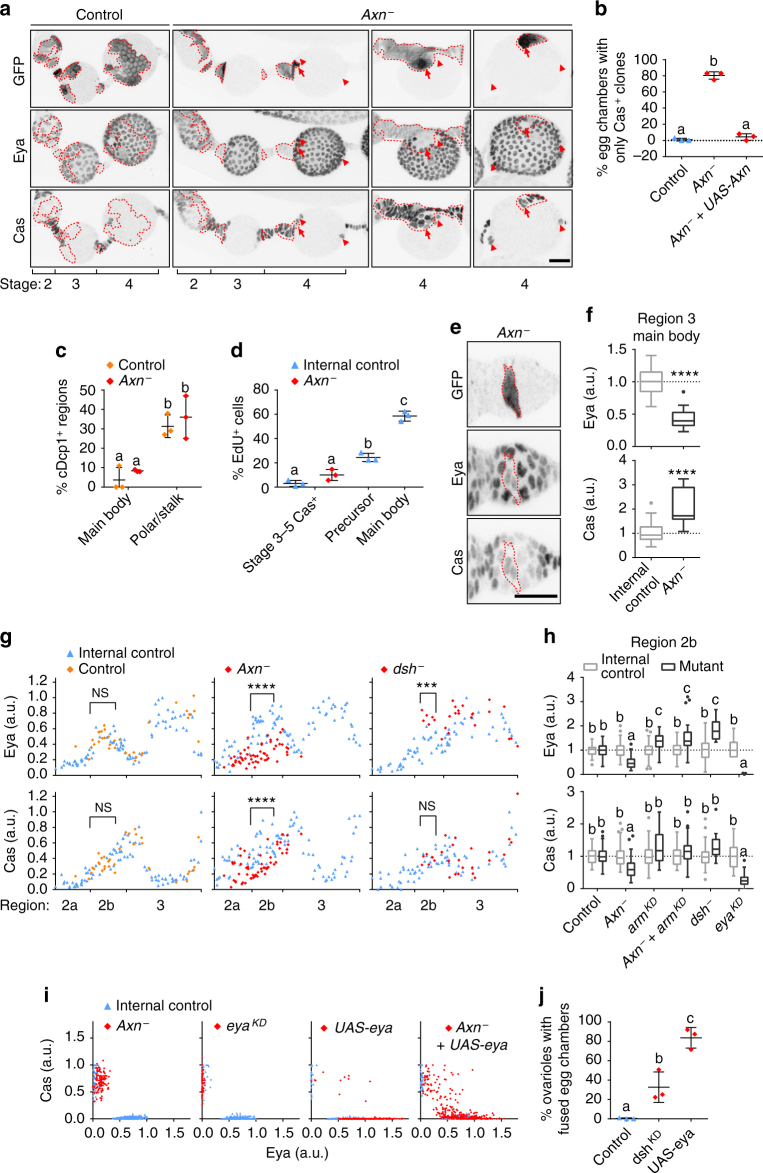
Wnt signalling inhibits expression of the main body cell fate factor Eya. **a** 3D projection view of one half of ovarioles with FRT82B control or *Axn* ^*S044230*^ mutant (GFP^+^, dashed lines) FSC clones. Arrowheads point to control polar cells in stage 4 of *Axn* ^−^, arrows point to the mutant polar cells. Eya^+^ GFP^−^ cells appear in the outlined stage 4 *Axn* ^−^ clones due to Z stack projection. **b** Stage 3–5 egg chambers with Cas^+^ only clones. Data (mean ± s.d.) from 3 experiments, 77-89 egg chambers per genotype. *P* < 0.0001. **c** Main body or polar/stalk regions with cells expressing cDcp1 in region 3-stage 2 FSC clones. Data (mean ± s.d.) from 3 experiments, 24-36 egg chambers. *P* < 0.05. **d** EdU incorporation in *Axn* ^*S044230*^ heterozygous control or homozygous mutant. Data (mean ± s.d. from three experiments, 446–812 stage 3–5 Cas^+^ cells, 465 germarium region 2b control cells, 1503 stage 3 main body control cells, *P* < 0.01. **e** 3D projection view of one half of a stage 1 egg chamber with *Axn* ^*S044230*^ FSC clones. **f** Eya and Cas intensity in region 3 main body cells. Data (median with interquartile range) from three egg chambers, 17–59 cells. Data were normalized to average Eya or Cas in internal control cells. **g** Eya and Cas intensity in germaria with FRT82B control (orange diamonds), *Axn* ^*S044230*^, or *dsh* ^*3*^ mutant clones (red diamonds) compared to control cells in the same germarium (blue triangles). **h** Eya and Cas intensity in germarium region 2b. Data (median with interquartile range) from 33–100 cells from 4 germaria per genotype. *P* < 0.01. **i** Eya and Cas intensity in stage 4 follicle cells. 1365–1434 cells from 4 stage 4 egg chambers per genotype. **j** *c* *306-Gal4*-driven *dsh* knockdown or *UAS-eya* overexpression in follicle precursor cells. Data (mean ± s.d.) from 3 experiments, 213-252 ovarioles. *P* < 0.05. Scale bars, 20 μm. ***P < 0.001; ****P < 0.0001 (Mann–Whitney test). Samples labelled with different letters are significantly different using ANOVA with Turkey’s test **b**–**d**, **j**, or Kruskal–Wallis with Dunn’s test **h**

*Axn* ^−^ FSC clones frequently gave rise exclusively to Cas^+^ cells (Fig. 4a, b). *Axn* ^−^ clones appear in the normal polar/stalk region, or as small clones within the main body forming ectopic polar and stalk cells, or as large clones that form a continuous stalk with a single polar cell cluster, causing the egg chamber to appear to bud from the side. Clones generated at a later stage, however, did not show this cell fate bias (Supplementary Fig. 7 ^15^), suggesting a narrow developmental time window for Wnt signalling to affect cell fate. To understand how hyper-activation of Wnt biased cells towards Cas^+^ polar/stalk-like fates, we considered a few possibilities. The *Axn* ^−^ Eya^+^ main body precursors may not survive, or Cas^+^ polar/stalk-like cells may proliferate more. Alternatively, or in addition, more cells may adopt a polar/stalk-like fate than a main body fate.

During early stages of oogenesis, apoptosis was common in polar and stalk cells but rare in main body cells (Fig. 4c ^35^). *Axn* ^−^ clones in region 3-stage 2 main body regions did not show a detectable increase in apoptosis (Fig. 4c). The mitotic marker 5-ethynyl-2′-deoxyuridine (EdU) showed a slight increase in *Axn* ^−^ Cas^+^ cells that did not rise to statistical significance (Fig. 4d) compared to control Cas^+^ cells in stages 3–5, suggesting that these polar/stalk-like cells did not proliferate more, consistent with previous observations^15^. Strikingly, we found a decrease of Eya and increase of Cas in the main body region in *Axn* ^*−*^ cells as early as region 3, suggesting a shift in cell fate (Fig. 4e, f). Such small clones in the main body region likely gave rise to ectopic Eya^−^ Cas^+^ clones observed in stage 4 egg chambers, whereas large main body clones likely developed into continuous stalks with a single polar cell cluster, causing the egg chamber to appear to bud from the side (Fig. 4a).

To understand how Wnt signalling affects follicle precursor cells, we quantified changes in Eya and Cas levels in germarium region 2b (Supplementary Movies 2–4). Eya was significantly reduced in the *Axn* ^−^ clones (Fig. 4g, h), which activated Wnt signalling in region 2b^P^ to the level normally found in region 2b^A^ (Fig. 3c). Conversely, *dsh* ^−^ cells showed significantly increased Eya in region 2b to a level closer to region 3 main body cells (Fig. 4g, h). The Eya intensity in region 2b *dsh* ^−^ cells was on average 1.83-fold compared to control cells in region 2b and 0.84-fold compared to control cell in region 3, suggesting that Wnt signalling normally functions to inhibit Eya expression. Reducing Wnt signalling by *armRNAi* also resulted in increased Eya, relative to control (Fig. 4h). In double *Axn* ^−^ */armRNAi* cells, *arm* was epistatic to *Axn* as expected (Fig. 4h). Cas was also reduced in *Axn* ^−^ cells in region 2b, but less so than Eya. Cas was similarly reduced in *eya* knockdown cells in this region (Fig. 4g, h), so the reduction of Cas in *Axn* ^−^ could be a secondary consequence of Eya reduction.

Eya is a potent determinant of polar and stalk cell fates, and knocking *eya* down in mosaic clones caused all mutant cells to become Eya^−^ Cas^+^ in stage 4 egg chambers, which phenocopied *Axn* ^−^ (Fig. 4i). To test how important the reduction of Eya was for the fate change in *Axn* ^−^ cells, we expressed *UAS-eya* in *Axn* ^−^ clones. Eya expression restored main body cell fate to many cells (Fig. 4i; Supplementary Fig. 8a, b). Therefore, Eya is a key target of hyperactive Wnt signalling. Expression of Eya in *Axn* ^−^ clones also produced Eya^+^ Cas^+^ cells and Eya^−^ Cas^+^ cells, which were also observed when expressing *UAS-eya* alone in mosaic clones. Thus the imperfect rescue may have been due to variations in the timing and level of Eya expression in these experiments.

Wnt affects Eya expression only transiently in region 2b, so some *dsh* ^−^ polar and stalk cells form (Supplementary Fig. 9a, b). However, 0/75 polar/stalk units analyzed were composed entirely of *dsh* ^−^ cells compared to 6/82 in controls clones (*P* = 0.03 by Fisher’s exact test). When *dsh* was reduced by *c306-Gal4*-driven RNAi, which was expressed in a larger group of follicle precursor cells than in the mosaic clone experiments (Supplementary Fig. 9c), we observed 22–51% of ovarioles with at least one egg chamber fusion (Fig. 4j, Supplementary Fig. 9d), indicating a problem with producing the correct number of polar and stalk cells in the right location for egg chamber budding. *UAS-eya* overexpression led to an even stronger effect: 72–92% ovarioles with fused egg chambers as previously reported^27^.

Together these results show that the level of Wnt signalling that is normally present in region 2b^A^ of the germarium is capable of repressing the main body cell fate determinant Eya. In *Axn* ^−^ clones, persistent activation of Wnt signalling to the 2b^A^ level at later stages produces a strong bias towards polar and stalk cell fates. Nevertheless, polar and stalk cells can form in the absence of Wnt signalling, albeit less frequently than normal. Therefore, we conclude that Wnt signalling is a transient input into Eya expression and that there are normally multiple inputs into Eya expression and progenitor cell fates.

### Hh signalling controls the timing of differentiation

**Fig. 5:**
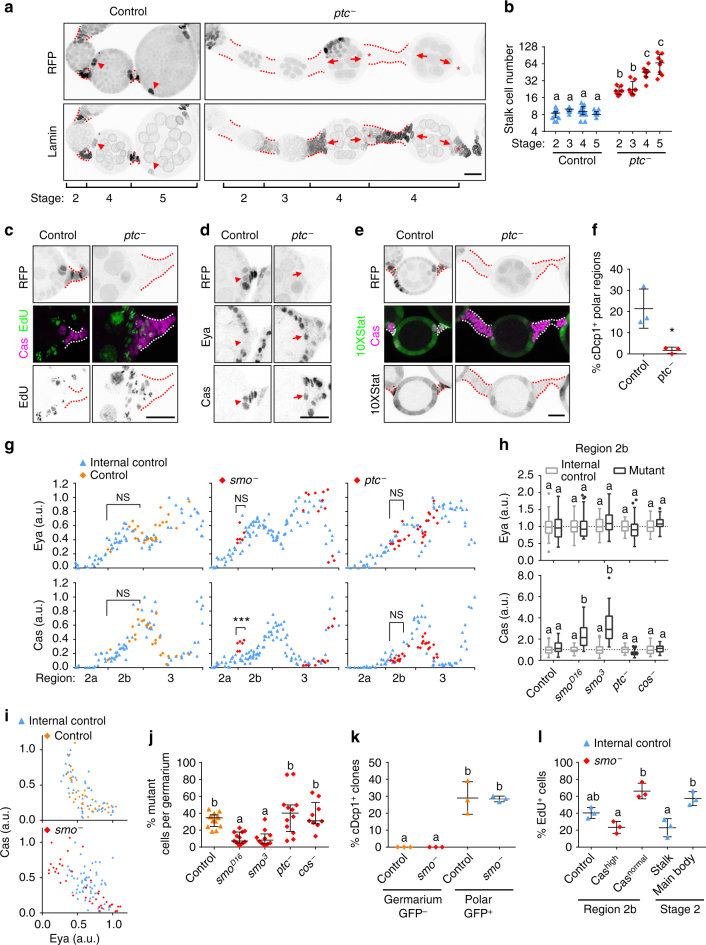
Hh signalling controls the timing of differentiation. **a** 3D projection view of ovarioles with *ptc* ^*S2*^ heterozygous control or large homozygous mutant (RFP^−^) FSC clones. Dashed lines mark the stalk region. Arrowheads point to control polar regions, arrows point to mutant regions. Asterisks marks mutant stalk cells proximal to posterior polar cells where Lamin is low. **b** Anterior stalk cell number in *ptc* ^*S2*^ heterozygous control or large homozygous mutant FSC clones. Data (median with interquartile range) from 8-15 egg chambers. *P* < 0.0001. **c, d** Sagittal view of posterior polar/stalk region in *ptc* ^*S2*^ heterozygous control or homozygous mutant egg chambers. **e** STAT activity shown by 10XStat-GFP in *ptc* ^*S2*^ heterozygous control or homozygous mutant stalk cell regions. **f** Clones with cDcp1 in *ptc* ^*S2*^ heterozygous control or homozygous mutant polar cell regions. Data (mean ± s.d.) from 3 experiments, 194-466 stage 2-5 polar cell regions. *P < 0.05 (Unpaired *t*-test). **g** Eya and Cas intensity in germaria with FRT40A control, *smo* ^*3*^, or *ptc* ^*S2*^ mutant clones compared to control cells in the same germarium. ***P < 0.001 (Unpaired *t*-test). **h** Eya and Cas intensity in germarium region 2b. Data (median with interquartile range) from 21 to 68 cells from 4-6 germaria per genotype. *P* < 0.001. **i** Eya and Cas intensity containing FRT40A control or *smo* ^3^ mosaic FSC clones in germarium region 3. Data from 85-105 cells from 3 germaria per group. **j** Mutant clone size. Data (median with interquartile range) from 9-13 germaria, *P* < 0.01. **k** Clones with cDcp1 in FRT40A control or *smo* ^3^ homozygous mutant clone (GFP^−^) in the germaria and GFP^+^ control cells in stage 1-2 polar/stalk regions. Data (mean ± s.d.) from 3 experiments, 39–51 ovarioles per genotype, *P* < 0.001. **l** EdU^+^ cells in *smo* ^3^ heterozygous control or homozygous mutant. Data (mean ± s.d.) from 3 experiments, 1222, 112, 119, 196, and 719 cells, *P* < 0.01. Scale bars, 20 μm. Samples labelled with different letters are significantly different using ANOVA with Turkey’s test **b**, **k**, **l**, or Kruskal–Wallis with Dunn’s test **h**, **j**

If hyperactive Wnt signalling causes excess and ectopic polar and stalk-like cells by inhibiting Eya and biasing cell fate, how does hyperactive Hh signalling cause both excess (Fig. 1e; Fig. 5a) and ectopic (Fig. 2e; Supplementary Fig. 10a ^32, 36^) polar and stalk-like cells while still producing main body cells (Figs 2e and 5a; Supplementary Fig. 10a)

We first analyzed the long stalk phenotype. The supernumerary stalk cell phenotype appeared most obvious when the majority of follicle cells associated with an egg chamber were mutant. In contrast to control stalks that contained a stable number of stalk cells ranging from 6 to 13, *ptc* ^−^ stalks contained an average of 21 cells at stage 2, and continued to increase in number over time (Fig. 5a, b). Another feature of stalk cell maturation is that they normally become physically separated from the polar cells (Fig. 5a, arrowhead), although stalk cells initially form at the poles. In contrast, *ptc* ^−^ stalk cells remained associated with the poles, and with Cas^+^ or Lamin C^+^ (another stalk cell marker^15^) cells on the main body (Fig. 5a, arrow). Mature Cas^high^ stalk cells are normally EdU-negative after stage 2 (Fig. 5c). In ovarioles with *ptc* ^−^ clones in regions where stalk was still connected to polar and/or main body cells, many mutant cells were EdU positive (Fig. 5c), and Eya and Cas were sometimes co-expressed (Fig. 5d), suggesting that some of the *ptc* ^−^ cells were multipotent precursors. Their low Lamin C expression level (e.g., asterisk in Fig. 5a) also resembled that of control precursors in the germarium. Therefore, excessive stalk cell production in *ptc* ^−^ clones seems due to persistence of precursor cells that continue contributing cells to the enlarging stalk.

We then asked how supernumerary polar cells form in response to Hh hyper-activation. Typically 3–6 polar cells form and all but two are eliminated by apoptosis^35^, a process that requires JAK/STAT signalling^37^. We observed a reduction of JAK/STAT activation in mutant polar cell regions (Fig. 5e), and reduced apoptosis of polar cells at stages 2–5 (Fig. 5f). Furthermore, some *ptc* ^−^ cells in the main body region became Eya^low^ Cas^high^ (Supplementary Fig. 10a), and thus formed ectopic polar/stalk cells as they matured, also consistent with the idea of a differentiation delay.

If hyperactive Hh signalling causes delayed differentiation, loss of Hh signalling should expedite differentiation. Earlier studies have not detected a differentiation defect in *smo* ^−^[21], but these studies did not have access to early cell fate markers and quantitative imaging. Using quantitative analysis of Eya and Cas in the germarium (Supplementary Movies 5–7), we observed that a subset of *smo* ^−^ cells showed significantly higher Cas expression in region 2b, or higher Eya expression in region 3 (Fig. 5g–i). The *smo* ^−^ clone size was significantly smaller than *ptc* ^−^ or control in the germarium (Fig. 5j), which could be due to apoptosis, or to premature differentiation into polar/stalk-like cells, which stop dividing early as part of their normal development. Cells with reduced Hh signalling in the germarium rarely showed any cDcp1 signal, although polar cell regions were frequently positive for cDcp1 as expected (Fig. 5k), suggesting that apoptosis was not the explanation. Interestingly, EdU incorporation in the Cas^high^ *smo* ^−^ cells was similar to the Cas^+^ stalk cells in stage 2, suggesting that a subset of *smo* ^−^ cells exited the cell cycle (Fig. 5l). Together, these results suggest that Hh signalling can suppress differentiation of follicle precursor cells.

We previously reported that some *smo* ^3^ clones lack Cas expression and thus polar and stalk cells^27^. Consistent with this observation, we found that *smo* mutant polar and stalk cell clones occur less frequently than control polar and stalk cell clones and less frequently than *smo* mutant main body clones (Supplementary Fig. 10b, c). However, using multiple *smo* alleles, we also found that this effect is not fully penetrant, so some *smo* ^−^ clones express similar Eya and Cas as controls at stage 4 (Supplementary Fig. 10d, e). Since *ptc* mutant cells show delayed differentiation as opposed to the dramatic cell fate bias observed in *Axn* mutant cells, we conclude that the reduction in polar and stalk cells is likely an indirect effect secondary to defective follicle cell differentiation.

### Wnt and Hh double mutants show additive effects

**Fig. 6:**
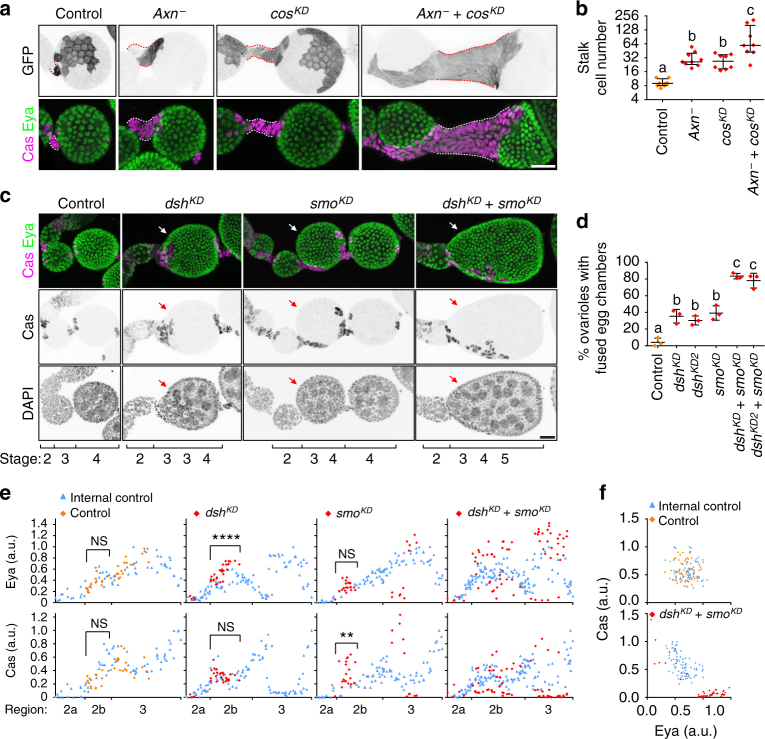
Wnt and Hh double mutants show additive effects on egg chamber patterning. **a** 3D projection view of one half of stage 4 egg chambers with FRT82B control, *Axn* ^*S044230*^, *cosRNAi*, or *Axn* ^*S044230*^+ *cosRNAi* mosaic FSC clones. Dashed lines mark the stalk region. **b** Quantification of stalk cells anterior to stage 4-5 egg chambers in FRT82B control, *Axn* ^*S044230*^, *cosRNAi*, or *Axn* ^*S044230*^ + *cosRNAi* mosaic FSC clones. Data (median with interquartile range) from *n* = 8–9 egg chambers per genotype. *P* < 0.01. **c** 3D projection view of ovarioles with stage 2–5 egg chambers in *c* *306-Gal4* control or *dshRNAi* and/or *smoRNAi*. Arrows point to fused egg chambers. **d** Quantification of fused egg chamber rate in c306-Gal4-driven knockdown of *dsh* and/or *smo* in follicle precursor cells. Data (mean ± s.d.) from *n* = 3 experiments, 145–189 ovarioles per genotype. *P* < 0.01. **e** Eya and Cas intensity in germaria with AyGal4 control (orange diamonds), *dshRNAi* and/or *smoRNAi* mutant clones (red diamonds) compared to control cells in the same germarium (blue triangles). **P < 0.01; ****P < 0.0001 (Unpaid *t*-test). **f** Eya and Cas intensity in germarium region 2b^P^ containing AyGal4 control or *dshRNAi* + *smoRNAi* mutant FSC clones. Data from 130–134 cells from 3 germaria per group. Scale bars, 20 μm. Samples labelled with different letters are significantly different using ANOVA with Turkey’s test **b**, **d**

If Wnt and Hh have distinct and independent effects on follicle precursor cell differentiation, hyper-activation or loss of both Wnt and Hh should show additive effects. Indeed, whereas a normal stalk contains 8–10 cells, we observed an average of 30 stalk cells in *Axn* or *cos* single mutants, and ~60 stalk cells in *Axn* and *cos* double knockdown cells (Fig. 6a, b). The *Axn* and *cos* double mutant cells are also largely biased towards Cas^+^ polar/stalk-like cells (Fig. 6a), suggesting that the Eya^+^ Cas^+^ precursors that accumulated due to delayed differentiation preferentially adopted a polar/stalk fate in the presence of high Wnt signalling. Loss of both Wnt and Hh also showed a more extreme phenotype than either alone. Combined loss of Wnt and Hh activity using *c306-Gal4* to drive *smo* and *dsh* RNAi in the follicle precursor cells increased the frequency of egg chamber fusions to 80% compared to 30–40% for the single knockdowns (Fig. 6c, d). In the germarium, double mutant cells showed signs of both increased Eya and premature differentiation into Eya^high^ Cas^low^ and Eya^low^ Cas^high^ cells (Fig. 6e, f; Supplementary Movies 8–11).

### Notch signalling promotes differentiation in multiple regions

**Fig. 7:**
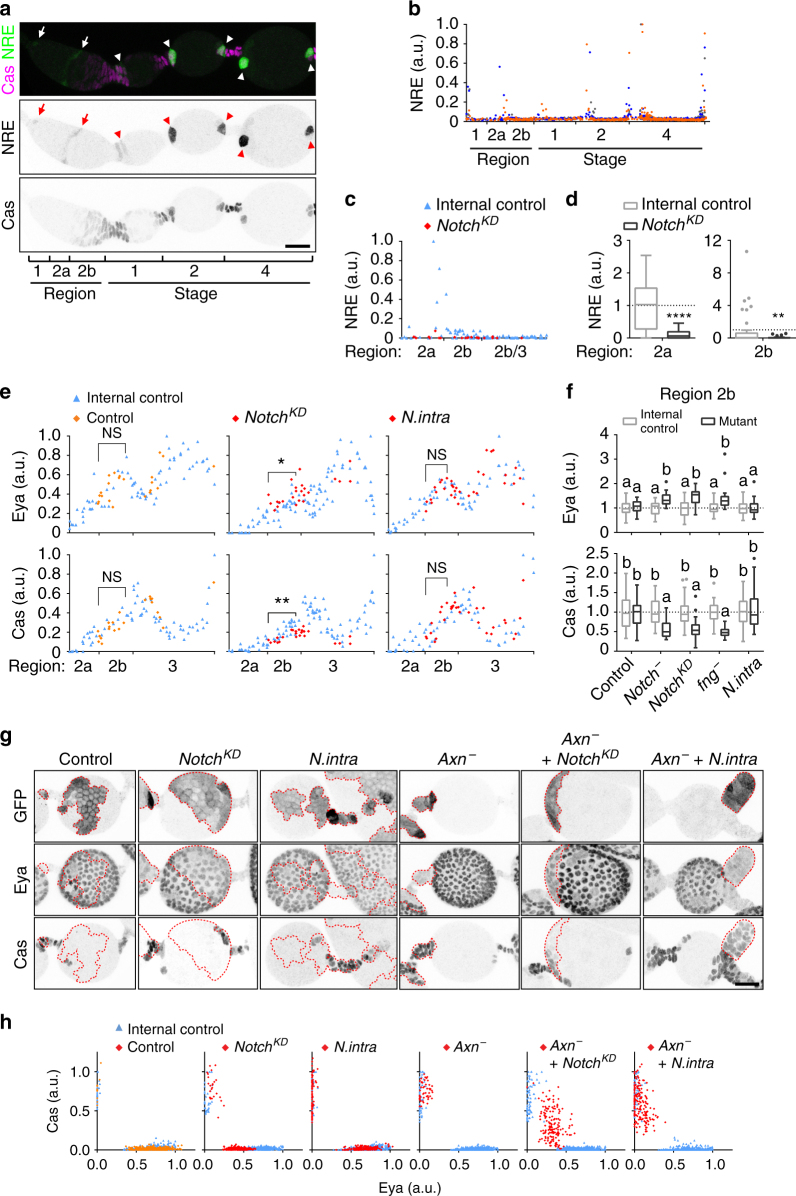
Notch is permissive but not instructive for differentiation of multiple cell types. **a** NRE-GFP pattern from the germarium to stage 4 egg chambers. Arrows point to Notch activity in the cap cells and region 2a/2b boundary cells and arrowheads point to polar cells. **b** Quantification of NRE-GFP intensity in cap cells, escort cells, and follicle cells along the anterior–posterior axis until stage 4. Data from *n* = 1533 cells in 3 ovarioles. Different colours represent different ovarioles. Data were normalized to maximum NRE-GFP intensity per sample. **c** NRE-GFP in germaria with *NotchRNAi* mutant clones (red diamonds) compared to control cells in the same germarium (blue triangles). **d** Quantification of NRE-GFP in germaria region 2a and 2b with mosaic clones. Data (median with interquartile range) from *n* = 20–66 cells from 3 germaria. Data were normalized to average NRE intensity in internal control cells. **P < 0.01, ****P < 0.0001 (Mann–Whitney test) **e** Eya and Cas intensity in germaria with FRT19A control (orange diamonds), *NotchRNAi*, or *UAS-N.intra* mutant clones (red diamonds) compared to control cells in the same germarium (blue triangles). *P < 0.05, **P < 0.01 (Unpaired *t*-test), **f** Quantification of Eya and Cas intensity in germarium region 2b containing FRT19A control, *Notch* ^*55e11*^, *NotchRNAi, fng* ^*13*^, or *UAS-N.intra* mutant FSC clones. Data (median with interquartile range) from *n* = 17-60 cells from 4 germaria per genotype. Data were normalized to average Eya or Cas in internal control cells. Samples labelled with different letters are significantly different at *P* < 0.01 (Kruskal–Wallis with Dunn’s test). **g** 3D projection view of one half of stage 4 egg chambers with FRT82B control, *NotchRNAi*, *UAS-N.intra*, *Axn* ^*S044230*^, *Axn* ^*S044230*^ + *NotchRNAi*, or *Axn* ^*S044230*^ + *UAS-N.intra* mosaic FSC clones (GFP^+^, dashed lines). Eya^+^ GFP^−^ cells appear in the outlined *Axn* ^*S044230*^ clone due to *Z* stack projection. **h** Quantification of Eya and Cas intensity in follicle cells with FRT82B control, *NotchRNAi*, *UAS-N.intra*, *Axn* ^*S044230*^, *Axn* ^*S044230*^ + *NotchRNAi*, or *Axn* ^*S044230*^ + *UAS-N.intra* mosaic FSC clones. Data from *n* = 1051–1420 cells from 4 stage 4 egg chambers per genotype. Scale bars, 20 μm

The involvement of Wnt and Hh in follicle precursor cell differentiation prompted us to ask how they relate to Notch signalling, a pathway known to be involved in ovary development at multiple stages^8, 9, 38^. We used a Notch activity reporter, the *Notch responsive element* (*NRE*) driving expression of GFP^39^ to address this question. NRE-GFP showed a basal level in all cells, but peaked in a subset of cells, in the region 2a/2b boundary cells and in polar cells (Fig. 7a, b). This is consistent with the known roles of Notch in the formation of the germline stem cell niche^40, 41^, cross-migration of FSC daughters^8^, and polar cell specification^9, 42^. Although the 2a/2b boundary signal was observed in only a subset of ovarioles, we confirmed that it depended on Notch, using *NotchRNAi* flip-out clones (Fig. 7c, d, Supplementary Fig. 11). Next, we asked whether Notch activity changed upon loss or hyper-activation of Wnt or Hh. We did not observe any change in Notch activity in germarium region 2b *dsh* ^−^, *Axn* ^−^, or *smo* ^−^ clones although it was reduced in *ptc* ^−^ cells (Supplementary Fig. 12a, b). *Axn* ^−^ clones showed Notch activity comparable to control polar cells both at egg chamber termini and at ectopic locations that were directly contacting the germline, suggesting that high Wnt signalling predisposes cells to specification as polar cells by Notch (Supplementary Fig. 12c). Polar cells show patchy NRE expression as reported previously^43^. We found the same percentage of *Axn* ^−^ cells showing NRE (66%, *n* = 91 *Axn* ^−^ polar cells) compared to control polar cells (65%, *n* = 177 control polar cells). Consistent with the conclusion that Hh signalling prevents premature differentiation of follicle cells in the germarium, *smo* ^−^ cells show precocious Notch activity in region 2b/3 (Supplementary Fig. 12a, d), while *ptc* ^−^ cells show delayed Notch activity in the polar cell regions (Supplementary Fig. 12e).

To assess the effect of Notch signalling on follicle precursor development, we quantified the Eya and Cas levels in *Notch* ^−^ and *NotchRNAi* FSC clones (Supplementary Movies 12-13). Although we previously did not detect an obvious change in Eya or Cas in the germarium^27^, with our new quantitative measurements we detected a slight decrease in Cas and a minor increase in Eya in region 2b (Fig. 7e, f). The Notch receptor modifier *fringe* (*fng*) mutant^44^ showed a similar phenotype as loss of *Notch* (Fig. 7f). In region 3, *Notch* ^−^ cells showed higher Eya and lower Cas in the polar/stalk region (Supplementary Fig. 13a). The change of Eya and Cas is consistent with the role of Notch in promoting both precursor cross-migration and differentiation, as well as polar cell specification, and we observed a high frequency of fused egg chambers when *NotchRNAi* clones covered the anterior polar cell region (Supplementary Fig. 13b).

Although loss of Notch activity causes loss of polar cells and egg chamber fusion^45^, hyper-activation of Notch by expressing the *UAS-N.intra* did not alter Eya or Cas levels in region 2b (Fig. 7e, f; Supplementary Movie 14), or induce ectopic polar/stalk cells on the main body region in stage 4 egg chambers (Fig. 7g ^10, 12^), suggesting that Notch activity is not sufficient for polar cell specification. Hyper-activation of Wnt together with hyper-activation of Notch caused an even larger number of polar and stalk-like cells (12.5%), compared to hyper-activation of Wnt (3.6%) or Notch (5.5%) alone (Fig. 7h). We noticed that polar cells normally express less Cas than stalk cells (82 ± 13% vs. 100 ± 13% comparing 17 polar and 25 stalk cells in control stage 4 egg chambers, *P* < 0.001), and constitutive Notch activity in *Axn* ^−^ increased the number of cells expressing polar as well as stalk cell Cas levels (Fig. 7h). Hyper-activation of Wnt together with loss of Notch caused follicle cells to show Eya and Cas patterns typical of precursor cells in stage 1 (Figs 2b and 7h), suggesting that Notch is necessary for differentiation of polar and stalk-like cells specified by Wnt signalling.

Interestingly, Notch appeared to promote further differentiation in multiple cell types even in stage 2-8 egg chambers. For example, *NotchRNAi* in the posterior pole cell caused Cas^+^ cells to abnormally express Eya (Supplementary Fig. 13c, d). Conversely loss of Notch in main body cells reduced the Eya level in stage 4 when Eya is normally increasing. In both cases, the observation is consistent with defective differentiation[38]. Furthermore, later in development the Eya level normally decreases again in main body cells and *NotchRNAi* prevented this (Supplementary Fig. 13e). *NotchRNAi* expressing stalk cells did not show the normal intercalated morphology (Supplementary Fig. 13c, d). Together, these results suggest that Notch promotes differentiation in multiple stages and cell types.

## Discussion

When adult tissue stem cells divide asymmetrically to self-renew and produce a daughter cell that commonly becomes a transit-amplifying precursor, the stem cell might retain its character by virtue of its proximity to niche signals whereas the transient amplifying precursors might acquire their properties due to displacement from the niche. Alternatively, niche signals might continue to influence precursor cell fate and differentiation. Here we report a previously undescribed Axn mutant phenotype that reveals a potent effect of Wnt signalling on follicle cell fates and therefore implicates Wnt signalling in diversification of follicle cell precursor fates as they leave the niche. We found graded responses to both Wnt and Hh and distinct actions of these niche signals, which together influence the development of the transit-amplifying follicle precursor population. Combining earlier studies on Wnt and Hh in follicle stem cell maintenance^15, 16, 46^ with this study, Wnt and Hh resemble morphogens that specify FSC fate at high concentration, a multipotent precursor fate at a lower concentration, main body precursor fate in the region lacking Wnt and low in Hh signalling, and differentiated cell fates in the absence of both Wnt and Hh. This proposal is consistent with a recently published study proposing that escort cells, which reside in the domain of highest Wnt activity, depend on Wnt for their fate^47^.

Earlier studies reported egg chamber formation defects due to reducing the Wnt or Hh ligand levels^13, 15, 32^. However, recent studies show that Wnt and Hh in escort cells also affect germline differentiation^19, 20, 48–51^; therefore any defects from reduction of Wnt or Hh ligands could either be due to effects on the germline and/or follicle cells. We clarified this issue by reducing Wnt and Hh intracellular signalling components directly in follicle cells in mosaic clones, or by RNAi knockdown specifically in follicle precursor cells.

In the germarium, the follicle precursor cells in regions 2b and 3 contain both specified and unspecified cells^8^, yet previous studies lacked cell fate markers and quantitative methods to assess the influence of different signalling inputs. Our quantitative analyses of Eya and Cas distinguish cell states in region 2b, where both markers are low but increasing, which we propose identifies a precursor state. In region 3/stage 1, fates begin to diverge into Eya^low^ Cas^high^ cells capable of producing polar or stalk cells and Eya^high^ Cas^low^ cells likely committed to main body fate. This combination of markers not only provides a sensitive and detailed description of the FSC differentiation process, but also reveals early changes in the germarium that were not previously detected. First, alterations in Wnt activity affect the expression of Eya, a key main body fate determinant^23^. Second, reduction of Hh activity in *smo* ^−^ cells resulted in increased levels of Cas in region 2b, which we suggest reflects premature differentiation. Although the Eya level did not increase in region 2b smo^−^ cells, this is likely because of inhibition of Eya by Wnt in this location. Third, reduction of Notch activity caused cells in region 2b to express slightly more Eya and less Cas than control cells, providing a possible explanation for how Notch promotes polar cell fate.

We show that hyper-activation of Wnt signalling biases follicle cells to polar and stalk cell fates, while loss of Wnt signalling causes a weak bias against polar and stalk fates. Why does loss of negative regulators produce a stronger effect than loss of positive ones? One theoretical possibility is that loss of negative regulators produces an extremely high and non-physiological level of signalling. However, our reporter data show that Wnt activity in *Axn* ^−^ cells is within the normal range found in region 2b (Fig. 2c). A second possibility is that the levels and distribution of negative regulators might be important factors shaping the Wnt response in that region. Unfortunately, this cannot be tested because available antibodies against Axn are not sensitive enough to detect the endogenous protein. A third possibility, which is not mutually exclusive, is that other factors^52^ work together with Wnt to inhibit Eya expression and specify polar and stalk cell fates in normal development.

Our results together with earlier reports^21^ support the idea that the graded response to Hh in the germarium serves to postpone differentiation. In *ptc* ^−^ cells differentiation is postponed so long that when it finally does occur, it is in absence of Wnt. Most cells therefore acquire a main body fate but a few cells can seemingly randomly acquire a Cas^high^ polar or stalk-like cell fate, as observed in stage 4–7 *ptc* or *cos* mutant clones (e.g., Fig. 2e, g; Supplementary Fig. 7a). This propensity may also explain the observation that some *dsh* ^−^ cells adopt polar and stalk fates. In contrast, in the presence of high Wnt signalling, as in *Axn* and *cos* double mutant cells, most of the cells adopt polar/stalk-like fates (Fig. 6a, b).

We suggest two explanations for how Notch might promote polar cell fate in the germarium. First, Notch has been shown to promote cross-migration of one FSC daughter^8^. Our results suggest that this will cause the precursor to remain exposed to a high level of Wnt for longer and therefore to remain low in Eya compared to a posteriorly displaced precursor cell. Second, we suggest that Notch promotes differentiation, which leads to the observed increase in Cas expression in region 2b. Although essential for polar cell specification, hyper-activation of Notch is not sufficient since constitutive activation of Notch causes excess polar cells to form only at the two poles rather than on the main body region^10, 12^. This implies that additional information is required besides Notch activity for polar cell fate. Here we report that short-range Wnt signalling maintains the potential for polar and stalk fates. Notch activity then acts on Eya^low^ precursor cells to specify polar cells and polar cells in turn express Upd to induce stalk cell fate^9^ in competent (i.e., Eya^low^), neighbouring cells.

Open questions remain. It is not entirely clear at what stage(s) polar cell fates become specified^8, 9^. Since the clones we analyzed were made in FSCs, it is possible that fluctuations in Wnt signalling within the FSC as it produces precursor cells could influence their fates—alternatively or in addition to the spatially graded response that we measured. The presence of ectopic Eya^−^ Cas^+^ cells in the fused egg chambers caused by the combined knockdown of *dsh* and *smo* shows that the polar/stalk fate can develop in the absence of Wnt and Hh, suggesting either that the knockdown is incomplete, that other factors are present that can promote Eya^−^ Cas^+^ fates, or that in the absence of normal signals follicle cell fates are unstable and can randomly tip towards main body or polar/stalk. The high frequency of fused egg chambers in the double knockdowns does confirm the importance of these pathways for the normal spatial and temporal patterning of cell fates.

Wnt, Hh and Notch are common players in many adult stem cell systems including the skin, gut, and blood^53–55^, which all possess a transit-amplifying progenitor pool close to the stem cell niche. Our finding of the separable functions of Wnt, Hh, and Notch in precursor cell fate specification, differentiation, and apoptosis provides an integrated model for how multiple signalling inputs produce the appropriate numbers and types of differentiated cells. The additive effects of hyper-activation of multiple signalling pathways described here may have implications for other adult stem cells, including cancer stem cells^56^.

## Methods

### *Drosophila* genetics and mosaic clone induction

Fly strains used in this study are listed in Supplementary Table 1. Fly genotypes used in each experiment are listed in Supplementary Data 1. Stocks were maintained at room temperature. Crosses were initiated at room temperature and transferred to 25 °C at 2–3 instar larvae stage. For *c306-Gal4*; *tubGal80ts* experiments, adult female flies were transferred to 29 °C for 7–10 days after eclosion. Egg chamber stage was determined based on germ cell nucleus diameter listed in Supplementary Table 2.

Mosaic clones were generated using the FLP/FRT system. 8–9 newly eclosed adult female flies (1–2 days old) along with 8 males were collected in a vial with wet yeast paste (dry yeast and water 1:1.5) and dry yeast and kept at 25 °C. Flies were flipped without CO_2_ to a fresh vial daily until dissection, and heat shocked 2 days after collection. Males were added if <3 were present to ensure optimal ovary development. For making FSC clones up to stage 5, flies were heat shocked twice for 1 h, about 4 h apart, in a 37 °C water bath, and then were kept at 25 °C for 5–7 days before dissection. For RNAi knockdown experiments, flies were transferred to 29 °C after heat shock (except for *eyaRNAi* and *armRNAi*, which were kept at 25 °C). For making FSC clones up to stage 8, flies were kept for 6–8 days before dissection. For border cell clones in Fig. 1d, flies were heat shocked once for 30 min and kept for 4–5 days before dissection. For negative mosaic clones, we excluded the false clones due to damage that contain condensed Hoechst staining and diffused nuclei signals. Polar and stalk cells always show higher ubi-RFP/GFP signals and therefore the intensity is not an indication of twin spot in those regions. For MARCM clones, we observed some leaky GFP expression in follicle cells in stage 6 and later, likely due to actinGal4 being too strong in stage 6 and later such that tubGal80 was not able to suppress all Gal4 activities. Therefore, we only analyzed MARCM clones before stage 6.

### Immunostaining and EdU incorporation

Adult female ovaries were dissected in Schneider’s *Drosophila* medium (Thermo Fisher Scientific, Waltham, MA) with 20% fetal bovine serum and transferred to a 0.6 ml microfuge tube with 100 μl dissection medium. Ovaries were dissociated by pipetting up and down ~50 times using a 200 μl pipette set to 50 μl. Dissociation in this way causes random physical damage to the egg chambers^57^, but we found it more efficient than pulling ovarioles out of the muscle sheath using forceps, which causes more damage to the germarium or younger egg chambers. Ovarioles were immediately fixed for 20 min in 4% paraformaldehyde at 4 °C. After fixation, ovarioles were washed with PBS/0.4% Triton X-100 (PBST), and then incubated with primary antibodies overnight at 4 °C. The following day, ovarioles were washed with PBST before incubation in secondary antibody for 1.5–2 h. After removal of secondary antibodies, samples were stained with Hoechst for 20 min. Samples were washed in PBST before sorting in PBST. Sorting was conducted by using forceps under a dissection microscope to remove mature eggs and clustered ovarioles from a given sample for optimal mounting. Without sorting, mature eggs make it difficult to compress the sample, the germaria can be tilted, and clustered ovarioles often overlap each other rendering imaging difficult. After sorting, samples were stored in VECTASHIELD (Vector Laboratories, Burlingame, CA) at 4 °C.

The following antibodies were used in this study: chicken anti-GFP (1:2000, Abcam, Cambridge, UK; 13970) (used to amplify MARCM GFP, flip-out GFP, Ptc-pelican-GFP, and NRE-GFP, not used on negative mosaic ubi-GFPnls), rabbit anti-dsRed (1:1000, Takara Bio USA, Mountain View, CA; 632496) (used to amplify flip-out RFP and NRE-RFP, not used on negative mosaic ubi-RFPnls or Fz3-RFP), mouse anti-Eyes Absent (1:50-200, Developmental Studies Hybridoma Bank (DSHB), Iowa City, IA; 10H6, needs pre-absorption if staining is noisy), mouse anti-Fascillin III (1:50, DSHB 7G10), rat anti-E-cadherin (1:50, DSHB DCAD2), rabbit anti-Castor (1:5000, Ward F. Odenwald^58^), mouse anti-Armadillo (1:100, DSHB N27A1), mouse anti-Smoothened (1:4, DSHB 20C6), rat anti-Cubitus interruptus (1:10, DSHB 2A1), rabbit anti-cleaved *Drosophila* caspase 1 (1:200, Cell Signaling Technology, Danvers, MA; 9578), mouse anti-Notch intracellular domain (1:200, DSHB C17.9C6), and mouse anti-Lamin C (1:200, DSHB LC28.26).

For EdU incorporation, adult female ovaries were dissected in Schneider’s *Drosophila* medium with 20% fetal bovine serum and transferred to a microfuge tube with the dissection medium plus 40 μM EdU, and kept at room temperature on a shaker for 1 h. Ovarioles were then dissociated, fixed, and stained with primary and secondary antibodies as described above. Before staining with Hoechst, an EdU detection reaction was performed according to the manufacturer’s manual (Thermo Fisher Scientific).

### Imaging and image processing

Due to the spherical organization of the egg chambers, few follicle cells have their nuclei located on the same imaging focal plane. Therefore, we imaged the egg chambers in full *Z* stacks. Samples were mounted on a glass slide in VECTASHIELD (25 μl for early stage ovarioles, or 65 μl for stage 9/10) using a 22 mm × 40 mm cover glass, to ensure that the germarium was mounted flat, but not compressed, and that later stages were compressed to a consistent degree. All images were taken on a Zeiss LSM780 confocal microscope, using a 40×1.4 N.A. oil objective. *Z* stacks covering the entire germaria or ovarioles were taken with a 0.43 μm step size for germarium and ovarioles, or a 1 μm step size for border cell clusters. XY resolution is 0.14 μm for germaria, or 0.35 μm for ovarioles. Laser power corrections were applied by increasing the laser power as the objective scans from the top of the sample to the bottom of the sample, so that the signal on the bottom did not appear weaker than the top.

3D images were visualized in Imaris (Bitplane, South Windsor, CT), and annotated in Excel (Microsoft, Redmond, WA), to categorize the developmental stage, sample condition, mounting condition, imaging condition, clone location, and result interpretation. Developmental stage was determined as described above. Sample condition includes whether they were damaged, or still tightly packed in muscle sheath. Severely damaged egg chambers had an incomplete follicle epithelium and leaky germ cells, or large patches of follicle cells without nuclear stain. Mild damage caused a small patch of follicle cells to show condensed Hoechst staining, and diffused or reduced nuclei Eya, Cas, or ubi-GFP/RFPnls signal^57^. Samples with severe damage were not analyzed, and the damaged cells in a sample with mild damage were not included in the analysis. We preferred to analyze samples out of the muscle sheath, because their morphology was not affected by squeezing from neighbouring egg chambers. Samples tightly packed in muscle sheath were not used for intensity measurement because it was difficult to perform laser power correction. Mounting condition denotes if the sample was too compressed or too tilted. If the germarium was too compressed, the germline cysts were squeezed and it was difficult to perform 3D rotation as described below. If too tilted, laser correction became difficult. Imaging condition marks whether the image was taken with proper laser power correction. This was estimated by comparing the signal intensity of the top, middle, and bottom of the sample visually, and was quantified as described below. Clone location and result interpretation were listed to help summarize the results, draw conclusions based on the phenotype seen across multiple ovarioles, and select representative images for presentation.

Representative images were exported from Imaris using either Easy 3D view or slice view. Since different follicle cell nuclei were located on different focal planes, 2–5 μm *Z* stacks were used to show single follicle cell layers, while 12–25 μm *Z* stacks were used to show one half of the egg chambers. Exported images were rotated and cropped in Photoshop (Adobe, San Jose, CA). Single channel images were converted from a black background to a white background using Invert LUT function in Fiji^59^.

### 3D quantification

Image segmentation was performed using Imaris. First, samples were rotated using the Free Rotate function. Egg chambers were rotated so the polar cells aligned horizontally, with the anterior to the left. Germaria were rotated in two steps. The first step positioned region 2b cysts vertically in the Z-direction by placing an Oblique Slicer in the mid-sagittal section of the germaria, and performing free rotation to the orthogonal view of the oblique slicer. The second step rotated the germaria anterior to the left to place region 2b cysts vertically in XY-direction. Second, follicle cell nuclei were detected using the Spots function. For the germaria, a 2.5 μm diameter spot size was used for automatic spot detection in the channel with follicle cell nuclei signals. Spots were then manually edited so that each follicle cell was marked. Dividing, dying, or damaged cells showed clear signs, including condensed Hoechst staining and diffused, or reduced, nuclei Eya, Cas, or ubi-GFP/RFP signal, and were not quantified. The 2.5 μm spots were then used to create a masked channel, and automatic spots detection based on that channel was applied to create 1.75 μm spots, so that only the centre of the nuclei with a strong and even signal was used for quantification. For egg chambers, a 3.46 μm diameter spot size was used for automatic spot detection, followed by reduction to 1.75–2 μm. Third, background intensities were estimated by placing 8–12 1.75–2 μm spots in two *Z* planes in same region as the measured follicle cells. For Eya and Cas, background spots were placed in the germ cell cytoplasm, while for Wnt or Hh reporters they were placed in the region 2b germ cell nuclei. Fourth, accuracy of laser power correction was determined by selecting control cells at the top, middle, and bottom of the germarium or egg chamber in the same region, and comparing their signal intensities.

Data for spot position and channel mean intensity were exported from Imaris, and processed using MATLAB (MathWorks, Natick, MA) for background subtraction, comparison of top, middle, and bottom intensity, and normalization, and plotted using Prism (GraphPad, La Jolla, CA).

### Statistics and reproducibility

All fly crosses were repeated at least twice and ovary dissections and staining were repeated at least three times. The exact sample size (*n*) is listed in Supplementary Data 1, representing biological replicates. Sample size was not predetermined by statistical methods but we used prior knowledge to estimate minimal sample size. The experiments were not randomized. Investigators were not blinded except when counting the *c306-Gal4* fused egg chamber phenotypes.

Standard statistic tests were performed using Prism. Sample sizes were appropriately large with appropriate distributions. Unpaired *t*-test (two-tailed) was used for comparing two groups with similar variance as determined by *F*-test. Mann–Whitney nonparametric test (two-tailed) was used for comparing two groups with different variance. Ordinary one-way ANOVA, followed by Tukey’s multiple comparisons test, was used for comparing multiple groups with similar variance as determined by Brown–Forsythe test. Kruskal–Wallis nonparametric test, followed by Dunn’s multiple comparisons test, was used when the variance is significantly different among multiple groups. For box plots, the Tukey method was used for plotting whiskers and outliers.

### Data availability

The authors declare that all data supporting the findings of this study are available within the article and its supplementary information files or from the corresponding author upon reasonable request.

## Electronic supplementary material

Supplementary Information

Description of Additional Supplementary Files

Supplementary Data 1

Supplementary Movie 1

Supplementary Movie 2

Supplementary Movie 3

Supplementary Movie 4

Supplementary Movie 5

Supplementary Movie 6

Supplementary Movie 7

Supplementary Movie 8

Supplementary Movie 9

Supplementary Movie 10

Supplementary Movie 11

Supplementary Movie 12

Supplementary Movie 13

Supplementary Movie 14
