# Supplementary material for: Quantitative microscopy of the Drosophila ovary shows multiple niche signals specify progenitor cell fate
Source: Nat Commun. 2017 Nov 1;8:1244. doi: 10.1038/s41467-017-01322-9 (PMC5665863; doi:10.1038/s41467-017-01322-9)
Supplement: Supplementary file 1 — Supplementary Information [file 41467_2017_1322_MOESM1_ESM.pdf]

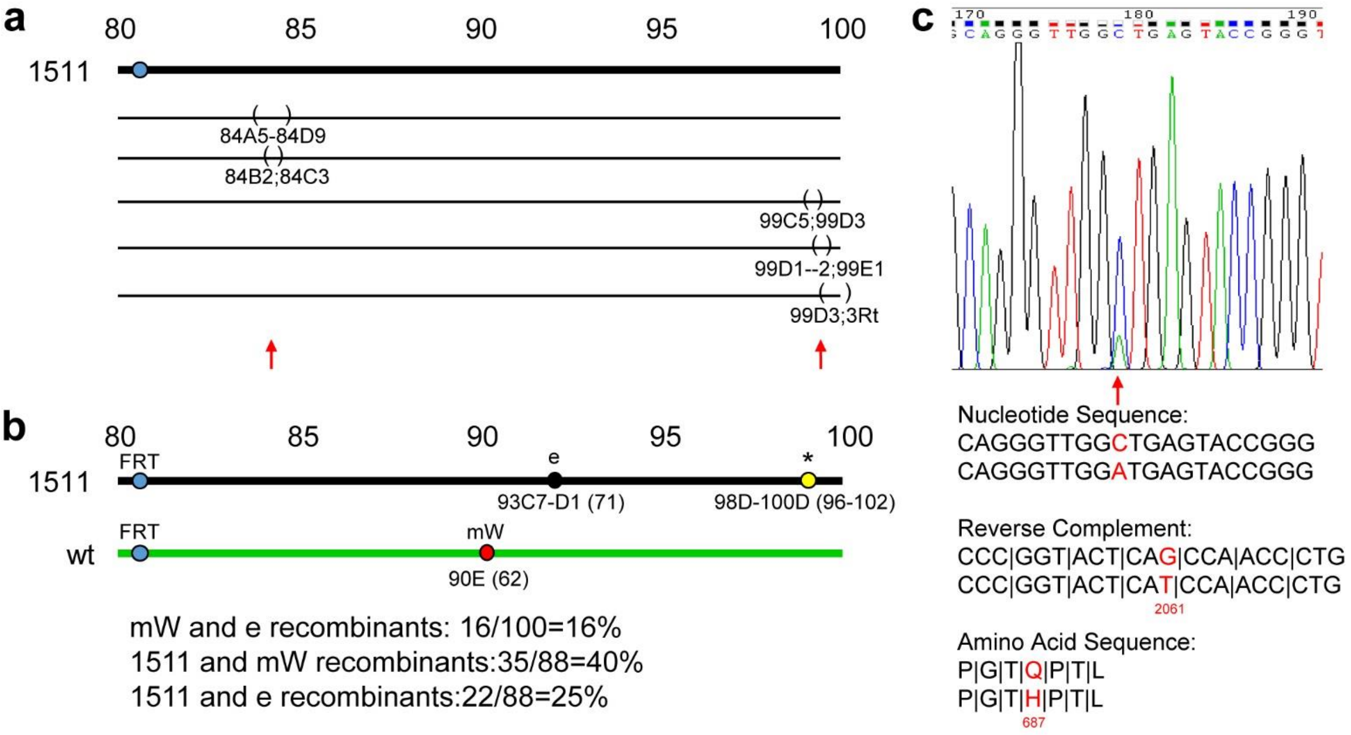

Supplementary Figure 1. Genetic mapping of *Axn*^{1511}.

(**a**) Mapping of *Axn*^{1511} by crossing to the chromosome 3R deficiency kit. Lethality was found when crossing 1511 to the 5 deficiency lines with the indicated deletions (brackets), which suggests that 1511 contains two lethal mutations (arrows). (**b**) Recombination mapping of *Axn*^{1511} by crossing to FRT82B, mW90E. The large border cell cluster phenotype (1511), red eye color (mW), and dark body color (e) were scored in recombinant lines. 1511 was mapped to chromosomal position 96-102 as estimated by recombination rates. The lethal mutation at chromosomal position 84 was removed in the recombinant used in Figure 1 and 2. (**c**) Point mutation detected by sequencing the coding sequence of the *Axn* genomic locus.

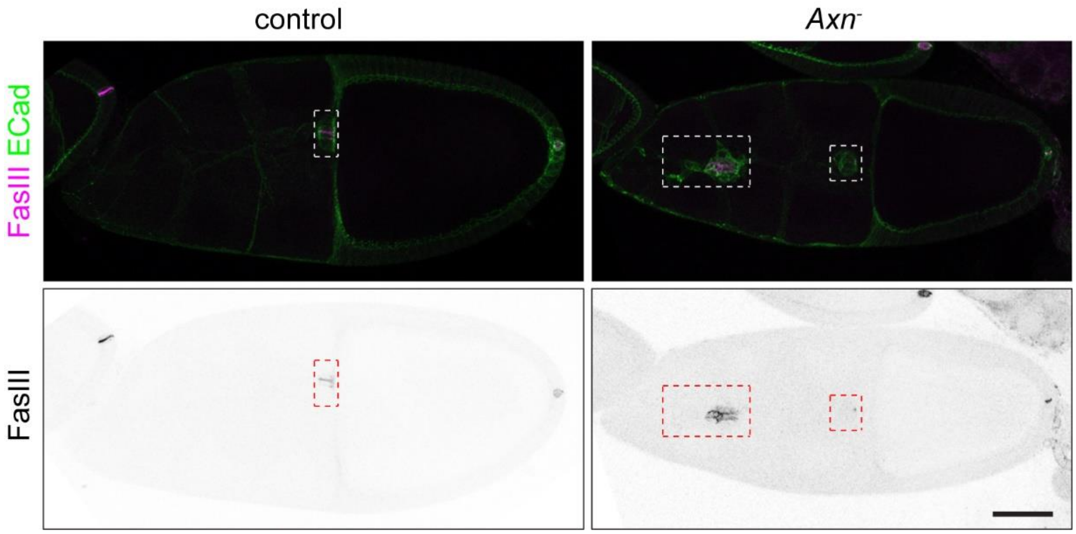

Supplementary Figure 2. ***$Axn^{S044230}$*** **mutant clones cause supernumerary polar cells.**

Border cell clusters (dashed boxes) in FRT82B control or FRT82B, *$Axn^{S044230}$* mosaic stage 10 egg chambers. ECad enriches in border cell clusters and FasIII accumulates on polar cell membrane. Scale bar, 50 μm.

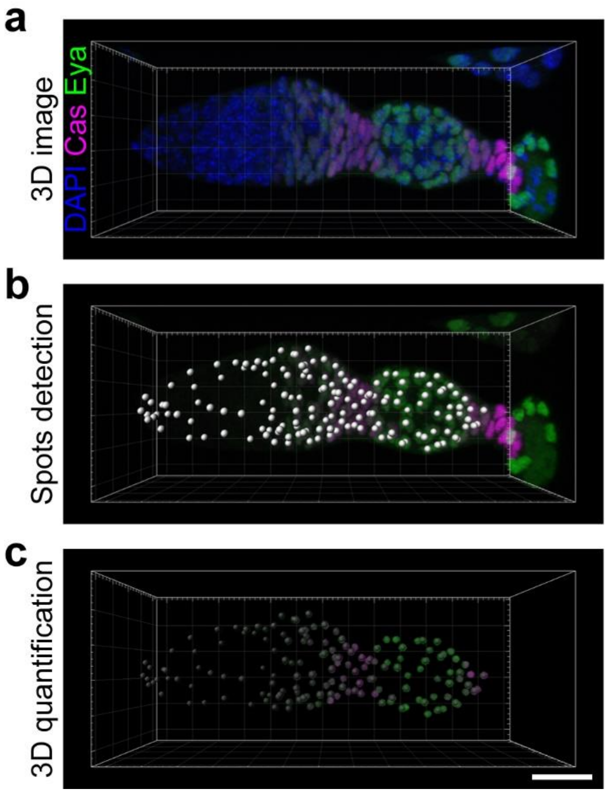

Supplementary Figure 3. Example of 3D quantification of the levels of both Eya and Cas in every somatic cell in a germarium.

(**a**) Image in 3D view. (**b**) Nuclei of somatic cells were first automatically detected by 2.5 µm spots (white dots) using the Eya channel, followed by manual proof editing using the Eya and DAPI channels to ensure one spot per nucleus. (**c**) A masked channel was created by setting the outside of the 2.5 µm spots to 0 intensity, and a 1.75 µm spot (semi-transparent dots) was automatically placed in the center of the 2.5 µm spot to get a strong and even nuclei intensity measurement. See methods for further details. Scale bar, 20 µm.

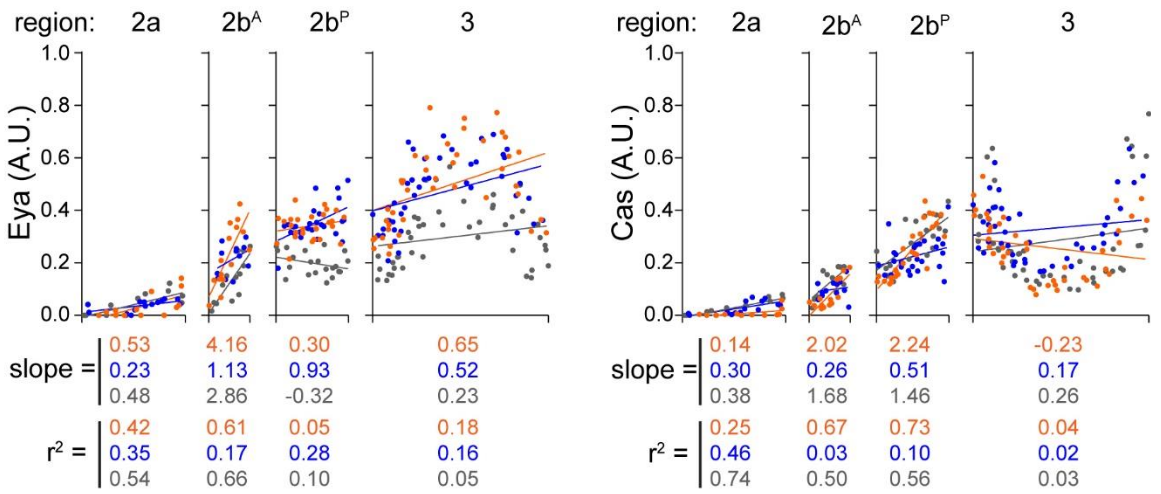

Supplementary Figure 4. Linear regression of Eya and Cas level in germarium along the anterior-posterior axis.

Quantification of Eya and Cas intensity in all somatic cells in germarium region 2a to region 3. Same data as shown in Fig. 2c. Different colours represent different germaria. Linear regression line as well as slope and R^2 are shown for each germarium.

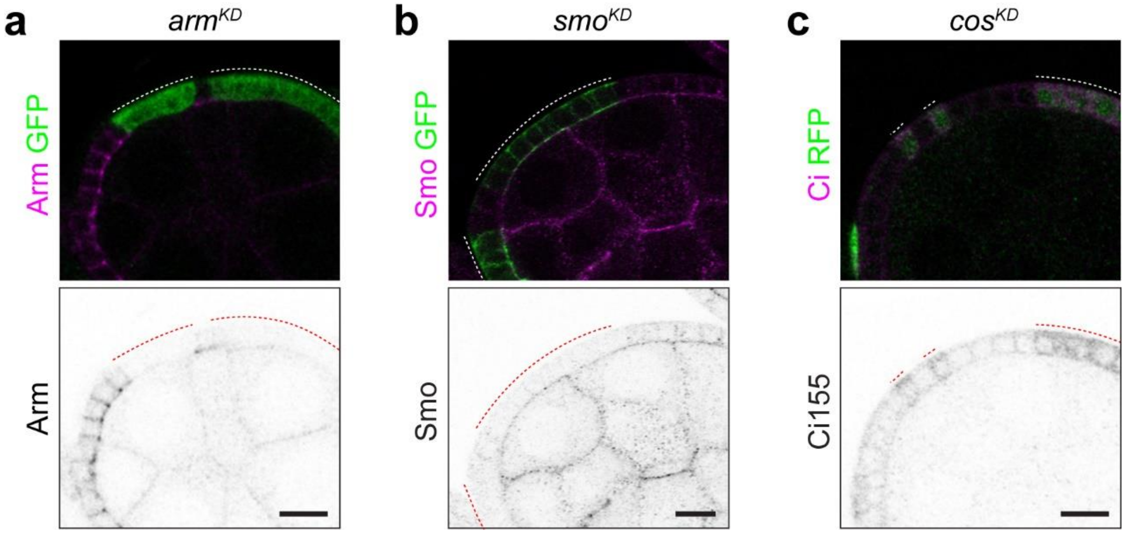

Supplementary Figure 5. Validation of RNAi lines used.

(**a**) Sagittal confocal section of *armRNAi* flip-out clones (GFP^+, indicated by the dashed line) in a stage 5 egg chamber stained with anti-Arm antibody (magenta in top panel, black in bottom). (**b**) Sagittal confocal section of *smoRNAi* flip-out clones (GFP^+) in a stage 6 egg chamber stained with anti-Smo antibody (magenta in top panel, black in bottom). (**c**) Sagittal confocal section of *cosRNAi* flip-out clones (RFP^+) in a stage 5 egg chamber stained with anti-Ci155 antibody (magenta in top panel, black in bottom). Scale bars, 10 μm.

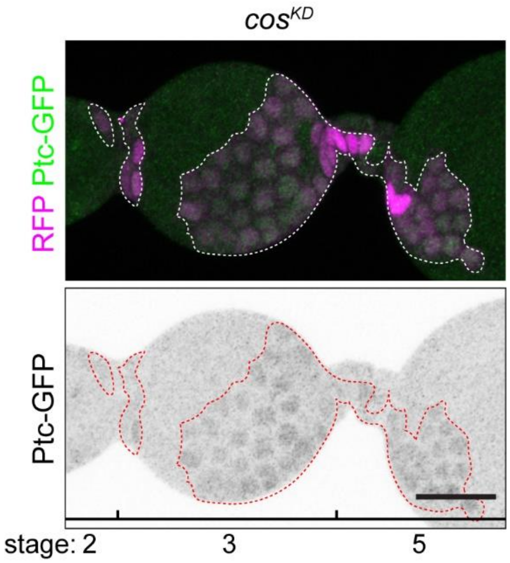

Supplementary Figure 6. ***ptc-GFP*** **signal in** ***cos^{KD}*** **ovariole.**

3D projection view of one half of stage 3-5 egg chambers with *cosRNAi* flip-out clones (RFP^{+}, dashed lines) showing the effect on expression of the *ptc-GFP* reporter. Scale bar, 20 µm.

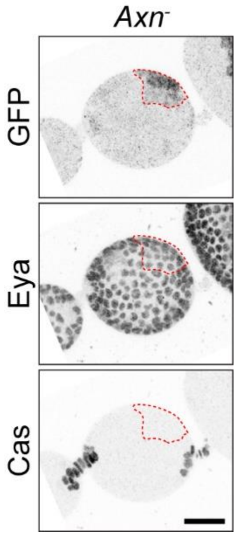

Supplementary Figure 7. ***Axn*^{S044230} transient clone does not bias towards polar/stalk fate.**

3D projection view of one half of egg chambers with *Axn*^{S044230} transient clones 2 days post clone induction (GFP^{+}, dashed lines) stained for Eya and Cas. Scale bar, 20 µm.

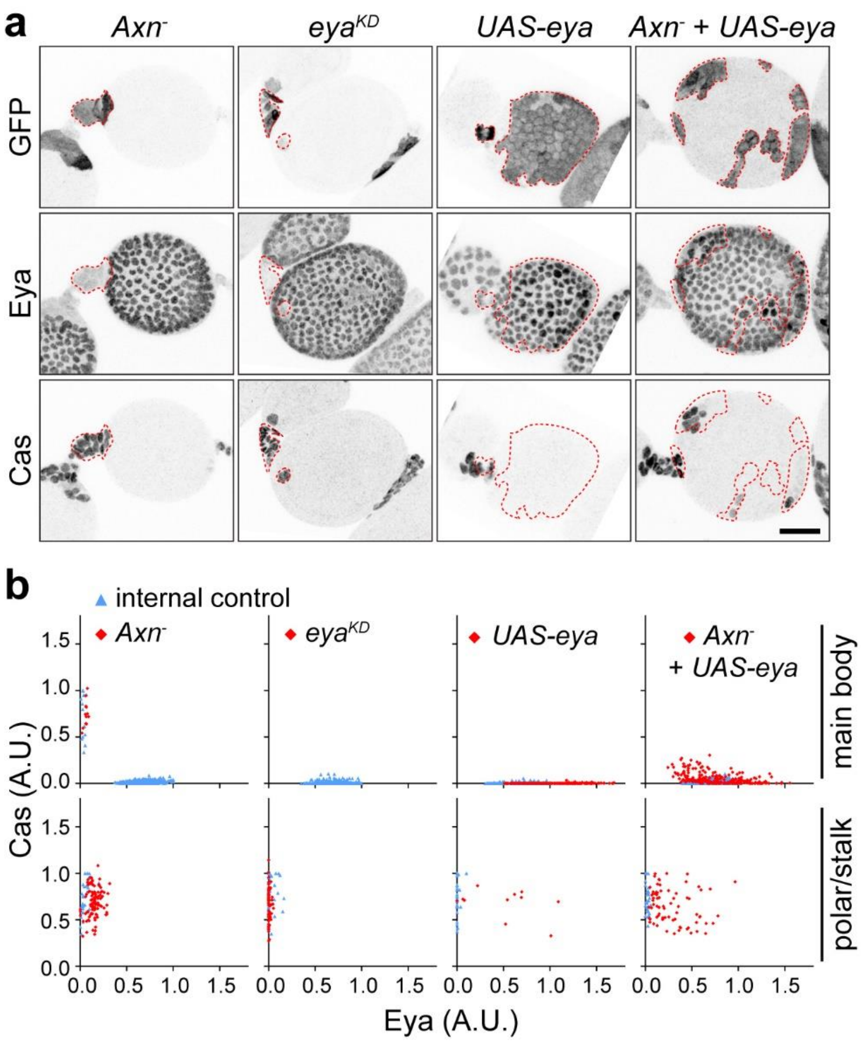

Supplementary Figure 8. Reduction of Eya is the key cause for the cell fate change in *Axn*⁻.

(**a**) 3D projection view of one half of stage 4 egg chambers with *Axn*^{S044230}*, eyaRNAi*, *UAS-eya*, or *Axn*^{S044230} + *UAS-eya* mosaic FSC clones (GFP^{+}, dashed lines marked mutant cells in the main body and anterior polar/stalk regions). Scale bar, 20 μm. (**b**) Quantification of Eya and Cas intensity in follicle cells in the main body or polar/stalk region with *Axn*^{S044230}*, eyaRNAi*, *UAS-eya*, or *Axn*^{S044230} + *UAS-eya* mosaic FSC clones. Data from n = 1,365-1,434 cells from 4 stage 4 egg chambers per genotype. A.U., arbitrary unit.

Supplementary Figure 9

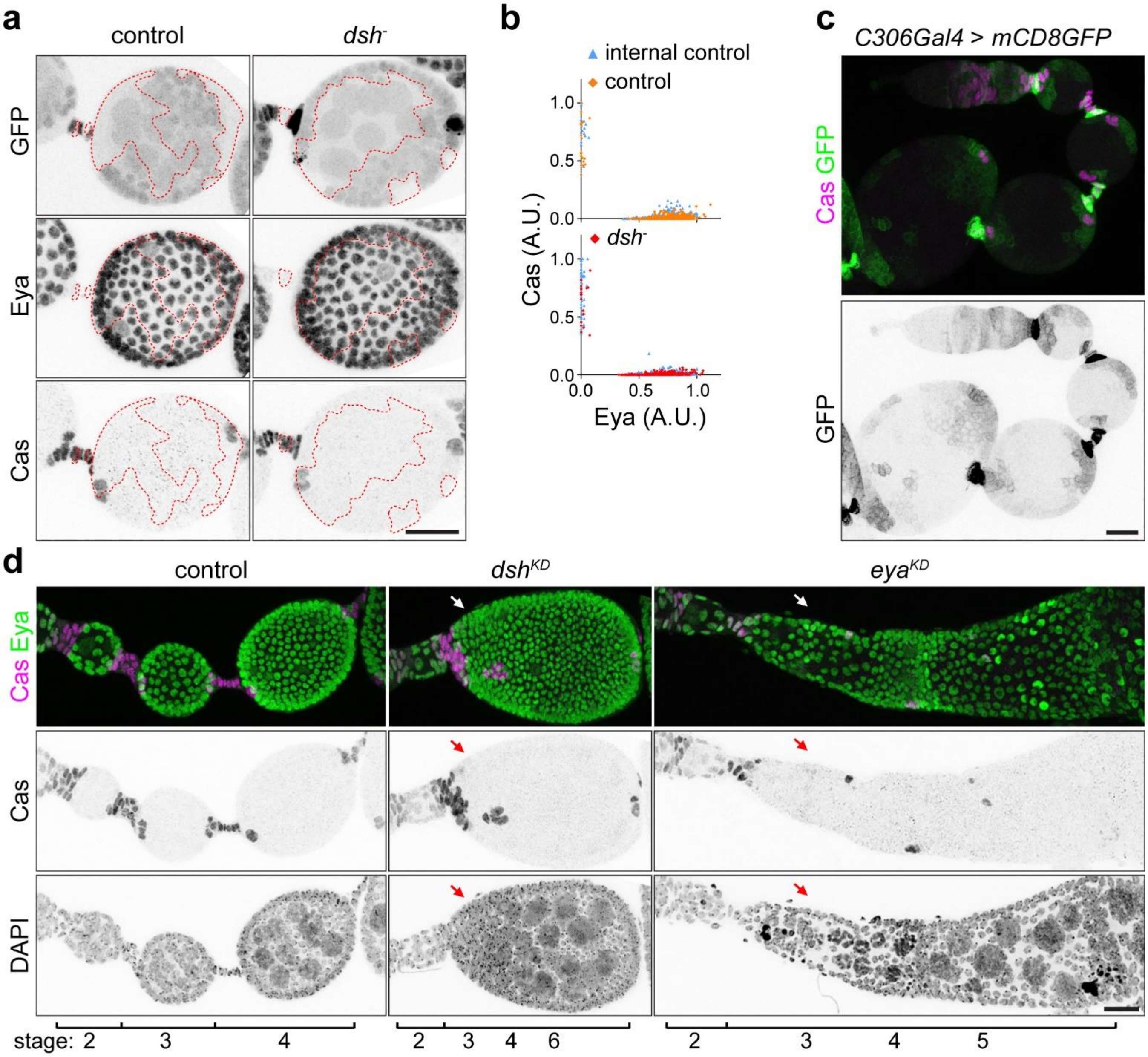

Supplementary Figure 9. Function of *dsh* in follicle precursor cells.

(**a**) 3D projection view of one half of stage 4 egg chambers with FRT19A control or dsh^3 mosaic FSC clones (RFP$^-$, dashed lines marked mutant cells in the main body and anterior polar/stalk regions). (**b**) Quantification of Eya and Cas intensity in follicle cells with FRT19A control or dsh^3 mosaic FSC clones. Data from n = 1,221-1,327 cells from 4 stage 4 egg chambers per genotype. A.U., arbitrary unit. (**c**) 3D projection view of an ovariole expressing *C306-Gal4* > mCD8GFP. (**d**) 3D projection view of ovarioles in *C306-Gal4* control, *dsh* knockdown or *UAS-eya* overexpression in follicle precursor cells. Arrows point to fused egg chambers. Scale bars, 20 µm.

Supplementary Figure 10

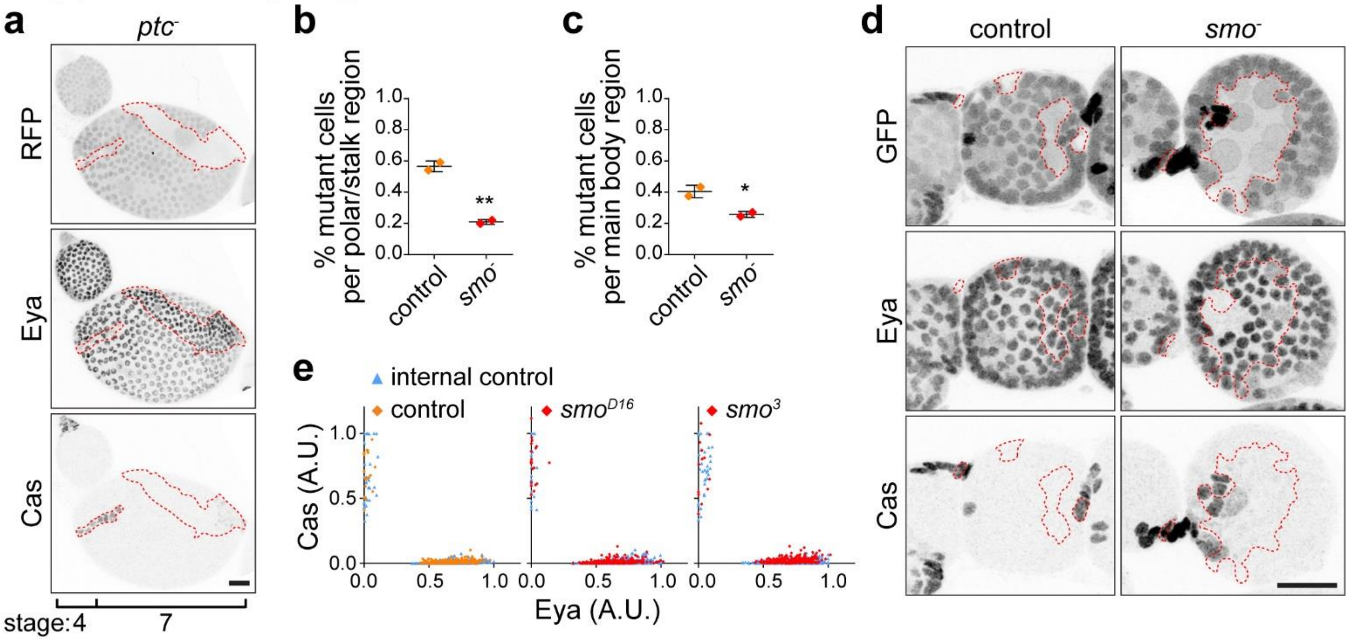

Supplementary Figure 10. Hh mutants in stage 4-7 egg chambers.

(**a**) 3D projection view of the follicle cell layer in *ptc*^{S2} clones (RFP$^-$, dashed lines) in a stage 7 egg chamber. Eya$^+$ RFP$^+$ cells appear in the outlined clone due to Z stack projection. (**b**) FRT40A control or *smo*^{3} clone percentage in stage 3-5 polar/stalk/polar units. Data (mean $\pm$ SD) from n = 2 experiments, 37-42 polar/stalk regions per genotype. $p < 0.01$. (**c**) FRT40A control or *smo*^{3} clone percentage in stage 3-5 main body cells. Data (mean $\pm$ SD) from n = 2 experiments, 37-40 egg chambers per genotype. $p < 0.05$ (Unpaired t test). (**d**) 3D projection view of one half of stage 4 egg chambers with FRT40A control or *smo*^{3} mosaic FSC clones (GFP$^-$, dashed lines marked mutant cells in the main body and anterior polar/stalk regions). (**e**) Quantification of Eya and Cas intensity in follicle cells with FRT40A control, *smo*^{D16} or *smo*^{3} mosaic FSC clones. Data from n = 1,066-1,306 cells from 4 stage 4 egg chambers per genotype. A.U., arbitrary unit. Scale bars, 20 μm.

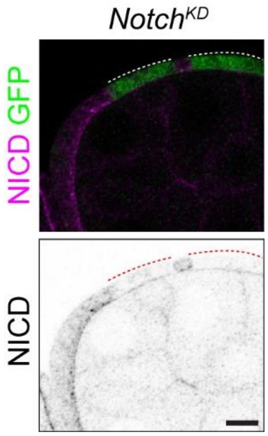

Supplementary Figure 11. Validation of *NotchRNAi*.

Sagittal confocal section of *NotchRNAi* flip-out clones (GFP^{+}) in a stage 6 egg chamber stained with anti- Notch intracellular domain (NICD) antibody. Scale bar, 10 µm.

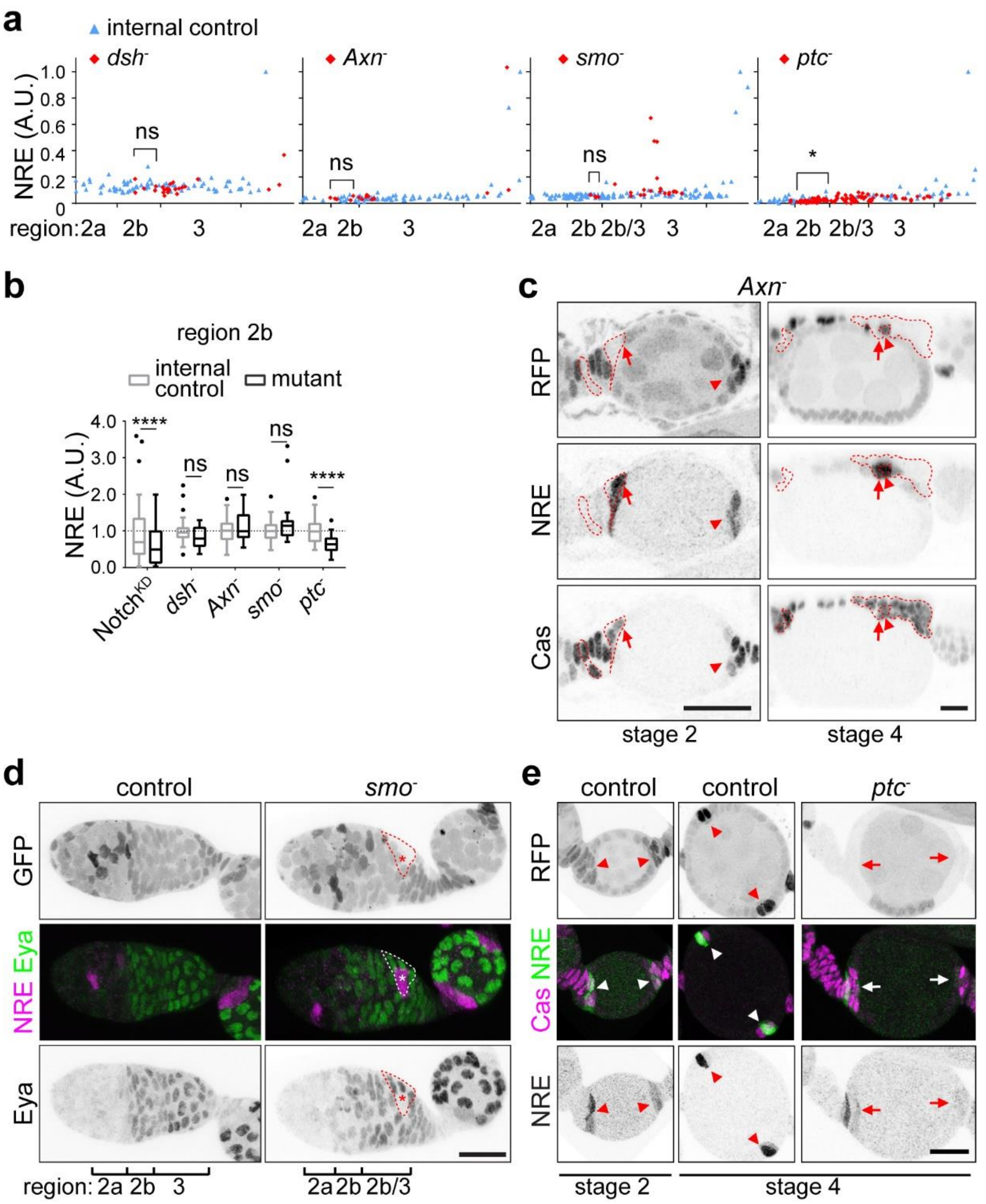

Supplementary Figure 12. Notch activity in loss or hyper-activation of Wnt or Hh.

(**a**) Notch reporter activity in germaria with *dsh*^{3}, *Axn*^{S044230}, *smo*^{3}*,* or *ptc*^{S2} mutant clones (red diamonds) compared to control cells in the same germarium (blue triangles). (**b**) Quantification of Notch reporter activity in germaria region 2b with mosaic clones. Data (median with interquartile range) from n = 15-64 cells from 3 germaria per genotype. Data were normalized to average NRE intensity in internal control cells. (**c**) NRE-GFP in *Axn*^{S044230} heterozygous control or mutant polar cell clusters in stage 2-6 egg chambers. Mutant cells are RFP^{-} (dashed lines). Arrowheads point to control polar cells, and arrows points to the mutant polar cells. (**d**) NRE-RFP in a *smo*^{3} mutant FSC clone in a germarium. Mutant cells are GFP^{-} (dashed lines), and the precocious Eya^{-}, NRE^{+}, presumptive polar cell is marked by *. (**e**) NRE-GFP in *ptc* ^{S2} heterozygous control (RFP^{+}, arrowheads) or homozygous mutant (RFP^{-}, arrows) polar cell regions. Scale bars, 20 µm. A.U., arbitrary unit. *, $p < 0.05$, ****, $p < 0.0001$ (Mann-Whitney test).

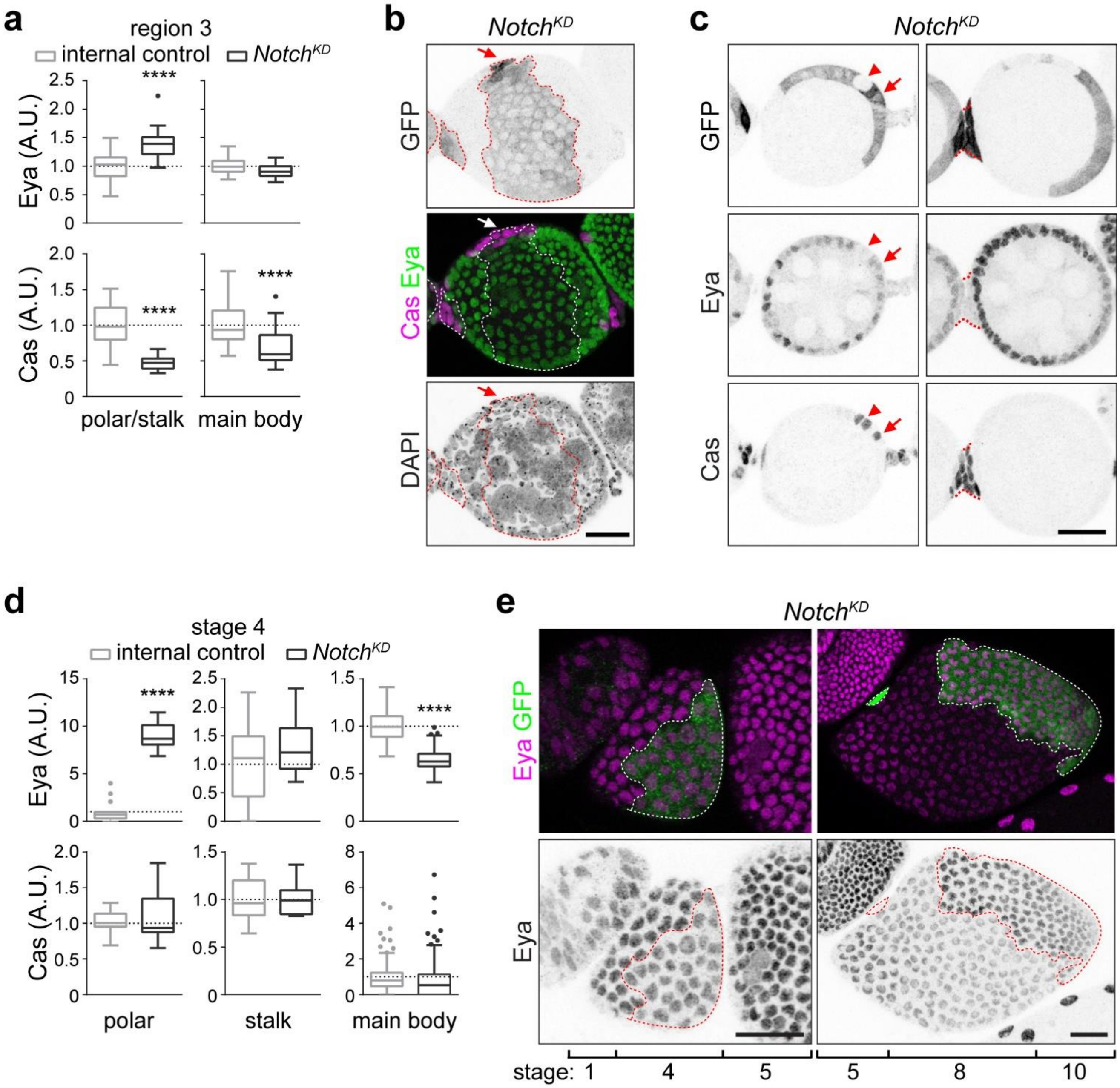

Supplementary Figure 13. ***NotchRNAi* effect on polar, stalk, as well as main body cell differentiation.**

(**a**) Quantification of Eya and Cas intensity in germarium region 3 containing *notchRNAi* mosaic FSC clones. Data from n = 21-37 cells from 3 germaria per region. Data were normalized to average Eya or Cas intensity in internal control cells. (**b**) *NotchRNAi* mosaic FSC clones (GFP^{+}) caused egg chamber fusion when the clone covered the anterior polar cell region (arrow). (**c**) Sagittal view of polar or stalk region in stage 4 egg chambers containing *NotchRNAi* mosaic FSC clones (GFP^{+}). Arrowheads point to control polar cells, and arrow points to the mutant polar cell. Dashed lines mark the mutant stalk region. (**d**) Quantification of Eya and Cas intensity in stage 4-5 polar, stalk and main body regions containing *NotchRNAi* mosaic FSC clones. Data from n = 9-19 polar or stalk cells or 105-132 main body cells from 3 egg chambers per region. Note for Eya level in polar cell plot, the control main body cell level is 30 ± 7 (mean ± SD). (**d**) 3D projection view of the follicle cell layer in *NotchRNAi* mosaic clones (GFP^{+}). Scale bars, 20 µm. ****, $p < 0.0001$ (Mann-Whitney test).

Supplementary Table 1: List of fly stains used in this study

Purpose	Genotype	Source	Reference
mutant	w;FRT82B,w+,Axn[1511]/TM3	outcross original mutant line FRT82B,*75G4,e/TM3,e to w; FRT82b,w+ and move the lethality allele at 84B2;84C3	1
	w;FRT82B,Axn[S044230]/TM3	Mark Peifer, remove 2nd chromosome balancers	
	w; FRT82B, Apc2[g10], Apc[Q8]/TM3	Mark Peifer, remove 2nd chromosome balancers	2
	w,dsh[3],FRT19A/FM7a	BDSC6331	
	w;smo[D16],FRT40A/CyO	Andreas Bergmann, remove FLP	
	yw;smo[3],y+,dp,FRT40A/CyO	lab stock, remove eya allele	
	y;FRT42D,ptc[S2]/CyO	BDSC6332	
	yw;FRT42D,cos2[H29],y+w-/CyO	Andreas Bergmann	3
	N[55e11],FRT19A/FM7c	BDSC28813	
	yw;fng[13],FRT80B/TM6B	BDSC8552	
mosaic driver and controls	ubi-RFPnls,w,hsFLP,FRT19A	BDSC31418	
	hsFLP,yw;ubi-GFPnls,FRT40A	combine BDSC1929 and BDSC5629	
	hsFLP,yw;FRT42D,ubi-RFPnls	combine BDSC1929 and BDSC35496	
	hsFLP,yw;GFP, FRT80B	combine BDSC26902 and BDSC5630	
	hsFLP,yw;FRT82B,ubi-RFPnls	lab stock	
	hsFLP,UAS-srcEGFP;actinGAL4,UAS-EGFP/CyO;FRT82B,armLacZ,tubGAL80/(MKRS)	Anna Jang	4
	hsFLP,yw;AyGal4,UAS-GFP (II)	combine BDSC1929 and BDSC4411	
	hsFLP;ActGal4(FRT.CD2),UAS-RFP/TM3	combine BDSC26902 and BDSC30558	
	hsFLP;AyGal4,UAS-moeGFP (III)	lab stock	
	yw,FRT19A	BDSC1744	
	yw;y+,FRT40A	BDSC1816	
	FRT42D;ry[605]	BDSC1802	
	w;FRT42D,w+	BDSC1928	
	w;FRT82B,w+	BDSC2050	
Gal4	C306Gal4;tubGal80ts	lab stock	
signaling reporter	w;fz3RFP (II)	Andrea Page-McCaw	5
	w;fz3RFP (III)	Erika Bach	5
	w;ptc-pelican/CyO	Todd Nystul	6
	w;NRE-pGR (II)	Sarah Bray	7
	w;NRE-pGR (III)	Sarah Bray	7
	w;NRE-pRR (III)	Sarah Bray	7
	w;10XStat-GFP (III)	BDSC26198	
fluorescent reporter and control	w;UAS-slbolifeactGFP (II)	lab stock	
	yw;UAS-mCD8GFP (II)	lab stock	
	w;UAS-GFPnls (III)	BDSC4776	
	w1118	lab stock	
UAS transgene	w;UAS-AxnV5	Yashi Ahmed	
	w;UAS-eya/(CyO)	Ilaria Rebay	8
	hsFLP;UAS-N.intra/(CyO);MKRS/TM2	BDSC52008	
RNAi	y,sr,v;eyaRNAi(57314) (II)	BDSC57314	
	yw;armRNAi(107344) (II)	VDRC107344	
	y,v;dshRNAi(31306) (III)	BDSC31306	
	y,v;dshRNAi(31307) (III)	BDSC31307	
	yw;cosRNAi(108914) (II)	VDRC108914	
	y,v;smoRNAi(62987) (II)	BDSC62987	
	w;NotchRNAi(1112)	VDRC1112	

Supplementary Table 2: Stage of egg chamber development

Stage**	Nurse cell nucleus diameter (µm)	
	anterior	posterior
2	5-7	
3	7-9	
4	9-12.5	
5	12.5-15.8	
6	15.8-18	
7	18-21.8	23-30
8	21.8-25	28-32
9	25-35	32-43
10	35-45	42-50

** Stage according to [9]

Supplementary References

1. Silver, D. L. & Montell, D. J. Paracrine signaling through the JAK/STAT pathway activates invasive behavior of ovarian epithelial cells in Drosophila. *Cell* **107,** 831–841 (2001).

2. Akong, K., McCartney, B. M. & Peifer, M. Drosophila APC2 and APC1 have overlapping roles in the larval brain despite their distinct intracellular localizations. *Dev Biol* **250,** 71–90 (2002).

3. Christiansen, A. E. *et al.* Non-cell autonomous control of apoptosis by ligand-independent Hedgehog signaling in Drosophila. *Cell Death Differ* **20,** 302–311 (2013).

4. Chang, Y. C., Jang, A. C., Lin, C. H. & Montell, D. J. Castor is required for Hedgehog-dependent cell-fate specification and follicle stem cell maintenance in Drosophila oogenesis. *Proc Natl Acad Sci U S A* **110,** E1734–42 (2013).

5. Olson, E. R. *et al.* Yan, an ETS-domain transcription factor, negatively modulates the Wingless pathway in the Drosophila eye. *EMBO Rep* **12,** 1047–1054 (2011).

6. Sahai-Hernandez, P. & Nystul, T. G. A dynamic population of stromal cells contributes to the follicle stem cell niche in the Drosophila ovary. *Development* **140,** 4490–4498 (2013).

7. Housden, B. E., Millen, K. & Bray, S. J. Drosophila Reporter Vectors Compatible with ΦC31 Integrase Transgenesis Techniques and Their Use to Generate New Notch Reporter Fly Lines. *G3 (Bethesda)* **2,** 79–82 (2012).

8. Hsiao, F. C., Williams, A., Davies, E. L. & Rebay, I. Eyes absent mediates cross-talk between retinal determination genes and the receptor tyrosine kinase signaling pathway. *Dev Cell* **1,** 51–61 (2001).

9. Spradling, A. C. in *The development of Drosophila melanogaster* 1–70 (Cold Spring Harbor Laboratory Press, 1993).
