# Supplementary material for: Quantitative microscopy of the Drosophila ovary shows multiple niche signals specify progenitor cell fate
Source: Nat Commun. 2017 Nov 1;8:1244. doi: 10.1038/s41467-017-01322-9 (PMC5665863; doi:10.1038/s41467-017-01322-9)
Supplement: Supplementary file 2 — Description of Additional Supplementary Files [file 41467_2017_1322_MOESM2_ESM.docx]

**Description of Additional Supplementary Files:**

File name: Supplementary Data 1

Description: List of fly genotypes and sample sizes in each experiment. () indicates the number of same region mutant and internal control cells, shown in brackets in the figure, that was used in statistical analysis.

File name: Supplementary Movie 1

Description: Animation of 3D reconstruction of a germarium stained with Eya, Cas, and DAPI. Germ cell cysts were detected using the DAPI channel. The steps of spot detection and quantification are indicated in the movie subtitles.

File name: Supplementary Movie 2

Description: Animation of 3D reconstruction of germaria containing FRT82B control (GFP^+^) mosaic clones stained with Eya, Cas, and DAPI.

File name: Supplementary Movie 3

Description: Animation of 3D reconstruction of germaria containing *Axn^S044230^* (GFP^+^) mosaic clones stained with Eya, Cas, and DAPI.

File name: Supplementary Movie 4

Description: Animation of 3D reconstruction of germaria containing *dsh^3^* (RFP^-^) mosaic clones stained with Eya, Cas, and DAPI.

File name: Supplementary Movie 5

Description: Animation of 3D reconstruction of germaria containing FRT40A control (GFP^-^) mosaic clones stained with Eya, Cas, and DAPI.

File name: Supplementary Movie 6

Description: Animation of 3D reconstruction of germaria containing *smo^3^* (GFP^-^) mosaic clones stained with Eya, Cas, and DAPI.

File name: Supplementary Movie 7

Description: Animation of 3D reconstruction of germaria containing *ptc^S2^* (RFP^-^) mosaic clones stained with Eya, Cas, and DAPI.

File name: Supplementary Movie 8

Description: Animation of 3D reconstruction of germaria containing AyGal4 control (GFP^+^) mosaic clones stained with Eya, Cas, and DAPI.

File name: Supplementary Movie 9

Description: Animation of 3D reconstruction of germaria containing *dshRNAi* (GFP^+^) mosaic clones stained with Eya, Cas, and DAPI.

File name: Supplementary Movie 10

Description: Animation of 3D reconstruction of germaria containing *smoRNAi* (GFP^+^) mosaic clones stained with Eya, Cas, and DAPI.

File name: Supplementary Movie 11

Description: Animation of 3D reconstruction of germaria containing *dshRNAi* + *smoRNAi* (GFP^+^) mosaic clones stained with Eya, Cas, and DAPI.

File name: Supplementary Movie 12

Description: Animation of 3D reconstruction of germaria containing FRT19A control (RFP^-^) mosaic clones stained with Eya, Cas, and DAPI. 3

File name: Supplementary Movie 13

Description: Animation of 3D reconstruction of germaria containing *Notch^55e11^* (RFP^-^) mosaic clones stained with Eya, Cas, and DAPI.

File name: Supplementary Movie 14

Description: Animation of 3D reconstruction of germaria containing *UAS-N.intra* (GFP^+^) mosaic clones stained with Eya, Cas, and DAPI.
